# The chicken gut virome: spatial structuring and extensive diversity of 19,778 viral populations

**DOI:** 10.1128/msystems.00191-26

**Published:** 2026-03-30

**Authors:** Johan S. Sáenz, Timur Yergaliyev, Bibiana Rios-Galicia, Jana Seifert, Amélia Camarinha-Silva

**Affiliations:** 1 Institute of Animal Science, University of Hohenheim 398728, Stuttgart, Germany; 2 HoLMiR—Hohenheim Center for Livestock Microbiome Research, University of Hohenheim 26558, Stuttgart, Germany; Quadram Institute Bioscience, Norwich, United Kingdom

**Keywords:** metagenomics, phages, chicken, gut

## Abstract

Viral communities, especially phages, affect prokaryotic diversity and thus influence the host’s metabolic processes. However, the makeup and role of the chicken gut virome remain poorly understood. To address this gap, we mined 1,458 chicken gut metagenomes and 56 viral-enriched samples to recover viral sequences and assemble a comprehensive collection of draft viral genomes. We identified 19,778 viral operational taxonomic units (vOTUs), of which 97% were dsDNA phages from the *Caudoviricetes* class, primarily targeting gut bacteria such as *Lactobacillus*, *Limosilactobacillus*, and *Escherichia*. Most protein-coding genes in these genomes were uncharacterized and lacked known biological functions. Additionally, the distribution of vOTUs across samples showed that the chicken virome is highly individual-specific. Yet, the viral community also exhibited strong spatial stratification along the gastrointestinal tract, with notable differences between proximal and distal regions, primarily driven by phages linked to the *Lactobacillaceae* family. Moreover, this study shows that the geographical region, breed, and diet drive the chicken gut viral diversity and composition. This underscores the significant novelty of the chicken gut virome and its largely unexplored functional potential, much of which would be missed if analyses were restricted to fecal samples.

**IMPORTANCE:**

The chicken gut harbors a vast community of viruses that remain largely unexplored despite their potential to influence poultry health and productivity. By analyzing 1,514 samples from different gut regions across 15 countries, we discovered nearly 20,000 distinct viruses, most of which were previously unknown phages. The chicken virome showed strong spatial differences along the gastrointestinal tract, meaning each gut section harbors a unique viral community, underscoring that fecal samples alone miss much of the virome’s diversity. We also uncovered that the geographical region, breed, and diet could drive the chicken gut viral diversity and composition. Overall, our findings greatly expand our understanding of gut virus diversity and microbiome ecology, offering a valuable foundation for developing strategies to monitor or manipulate the microbiome to improve poultry health.

## INTRODUCTION

The chicken gut is a complex and dynamic multi-kingdom ecosystem, where diverse microbial communities, including bacteria, archaea, fungi, and viruses, interact with the host and influence its physiology and productivity (1). The most abundant and well-studied members of the chicken gut microbiota are the bacteria *Lactobacillus*, *Limosilactobacillus*, *Bacteroides*, *Blautia*, *Ruminococcus*, and *Faecalibacterium*, which contribute to fiber degradation, short-chain fatty acids (SCFA) production, and the metabolism and binding of carbohydrates (2–5). While fungal communities have received less attention, genera such as *Candida* and *Aspergillus* have been identified in the chicken cecum, with *Candida* being frequently described as the most abundant genus (5, 6). In contrast, most studies of viral communities in chickens have focused on eukaryotic RNA viruses, with phages remaining largely overlooked (7, 8).

The chicken gut microbiome has been associated with the modulation of host health and production. For example, microbiota-derived metabolites like SCFA, amino acids, vitamins, and secondary bile acids contribute to enhancing the epithelial barrier, oxidative stress resilience, immune signaling, anti-inflammatory effects, energy balance, and metabolic efficiency (9–11). Similarly, studies in chickens show that microbial composition is linked with colonization resistance against pathogens such as *Salmonella* (12, 13). Additionally, the early colonization of chicken gut by bacteria promotes immune maturation, thereby increasing resistance to infection in older birds (14). From a production perspective, a more diverse microbiome has been associated with increments in growth rate (15), feed efficiency (16), and egg production (17). In this way, the chicken gut microbiota is an important interface linking nutrition, management, and production efficiency in modern poultry systems.

The chicken virome has been studied primarily in the context of eukaryotic viruses that can infect the animal and broadly affect animal health and production (18, 19). It is also recognized that eukaryotic viruses alter the gut microbial community and promote the proliferation of undesired bacteria, leading to various health problems (20, 21). Additionally, viruses seem to be acquired from farm environments and through contact with other animals, including those linked to gut diseases, underscoring the need to better understand their ecology (22). Phages, viruses that infect bacteria, are key regulators of microbial ecosystems, including those within the gastrointestinal tract. Through lytic and lysogenic cycles, phages exert top-down control over host populations, shaping community structure and dynamics (23). In the gut, such interactions influence host metabolism, immune modulation, and colonization resistance (24, 25). Even though phages from the chicken gut have been scarcely described, some studies using phage cocktails and fecal viral transplantation have shown that phages can modulate the chicken gut microbial composition, including the exclusion of pathogens (26, 27). This indicates that phages could have different potential applications in the management of poultry systems.

In recent years, efforts to explore the enormous microbial diversity have become possible through bacterial and archaeal metagenome-assembled genomes (MAGs) obtained from uncultured organisms. However, metagenomic samples have also been valuable for recovering extracellular and intracellular viral sequences. For example, extensive novel viral diversity has been found in the human gut (28), rumen (29, 30), soil (31), and ocean environments (32, 33). These novel viral genomes have contributed to untangling the complex viral taxonomy (34) and host-virus interactions, which could be exploited to develop phage therapies and modulate prokaryotic metabolism. Most metagenomic studies to date have relied exclusively on fecal samples, providing only a snapshot of viral communities in the distal gut. As a result, we still lack a comprehensive understanding of their distribution and ecological roles throughout the entire gastrointestinal tract. Extending viral metagenomics to distinct gut compartments can reveal spatial heterogeneity and environmental drivers of viral communities. Viral metagenomics across different compartments of the chicken gut could be crucial for addressing critical challenges in poultry production, such as preventing avian influenza, improving feed efficiency, and enhancing overall productivity to meet the growing global egg demand (35, 36).

Given the potential importance of viral communities in chicken gut health and productivity, we mined viral sequences from 1,514 chicken gut samples across 15 countries and three continents, spanning the crop, duodenum, jejunum, ileum, caeca, colorectum, and feces. This approach allowed us to collect 47,092 draft genomes and 19,778 species-level viral operational taxonomic units (vOTUs), thereby expanding the unknown diversity of DNA viruses in the chicken gut. Additionally, this data set reveals a strong spatial stratification along the chicken gastrointestinal tract, with marked differences between the proximal and distal regions, which is not captured solely by fecal samples. These findings improve our understanding of host-virus interactions and provide a valuable resource for studying the role of phages in the chicken gut.

## RESULTS

### A collection of DNA viruses from the chicken gut

**Fig 1:**
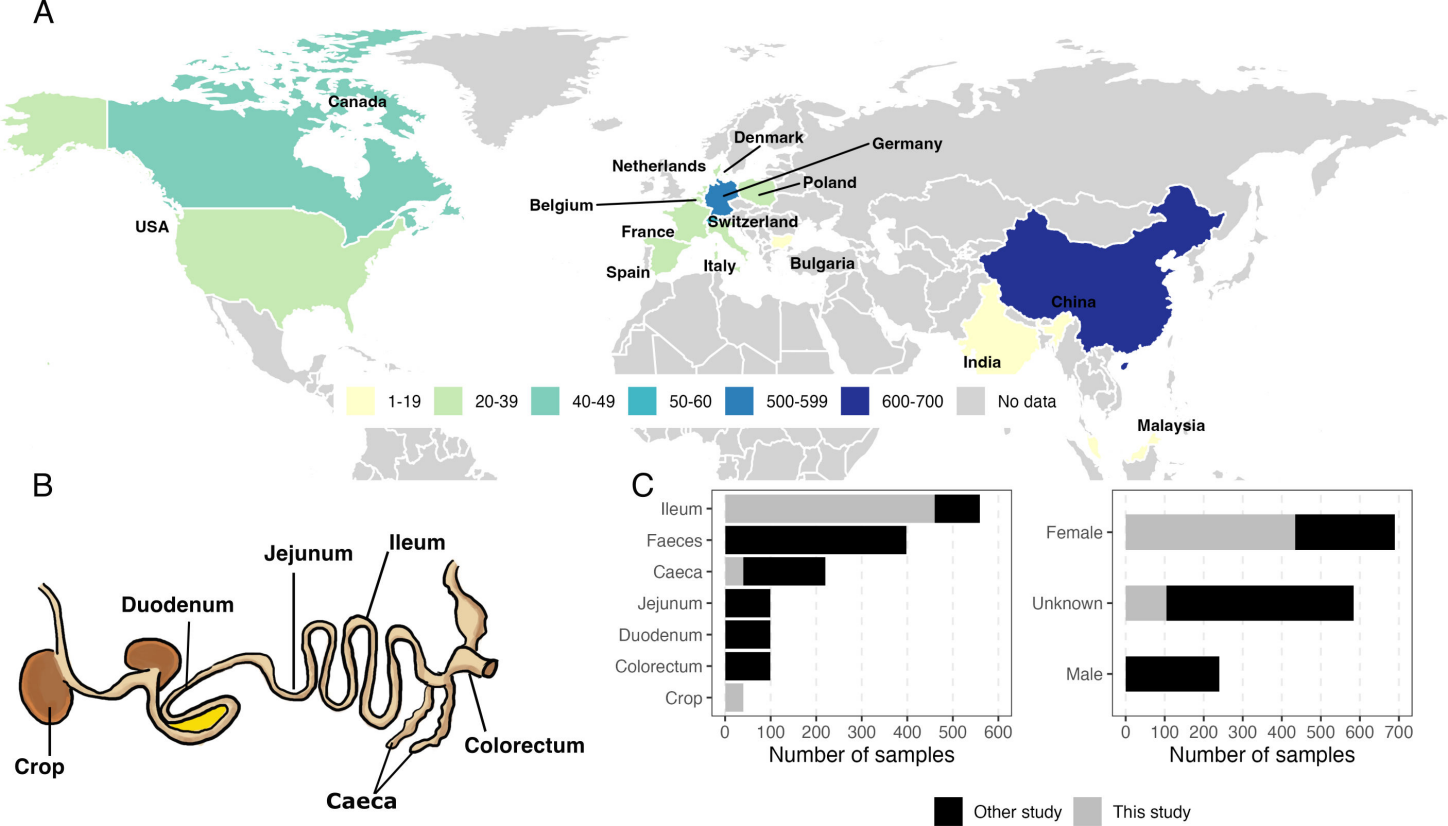
Global distribution and gastrointestinal origin of the collected 1,514 chicken gut metagenomic samples. (**A**) Map showing the distribution of collected samples across 15 countries. The map was created using R and the package ggplot2; the base map is from Natural Earth. (**B**) Schematic representation of the different chicken gut regions covered by the collected samples analyzed in this study. (**C**) Distribution of metagenomic samples by gut region and sex.

To create a collection of DNA viruses from the chicken gut and explore their diversity, we mined DNA viral sequences from 1,458 chicken gut metagenomic samples and 56 viral-enriched samples, representing approximately 5.5 TB of data. The data set comprised samples from independent studies conducted in 15 countries across Europe (*n* = 775), Asia (*n* = 663), and North America (*n* = 76) (Fig. 1A; Tables S1 and S2) (2, 37–42). The data set included samples from animals of different sexes and ages, collected from both feces and six distinct gut regions: crop, duodenum, jejunum, ileum, caeca, and colorectum (Fig. 1B and C). After quality controlling the raw reads and removing host DNA, samples were assembled using MEGAHIT (43), which yielded approximately 416 million contigs. To identify putative viral sequences, we applied the geNomad pipeline to contigs longer than five kbp (approximately seven million contigs in total), which revealed 246,000 single contigs that could potentially be considered viral sequences. The quality of viral draft genomes derived from assembled metagenomic samples can vary broadly, from low quality (< 50% complete) to near-complete or complete. We used checkV (44) to determine the completeness and contamination of the viral sequences, selecting 47,092 viral draft genomes (>40% completeness, further referred to as genomes). Among these, 7,178 were classified as complete genomes, identified through the presence of direct terminal repeats (*n* = 5,640), inverted terminal repeats (*n* = 1,491), and provirus boundaries (*n* = 47).

**Fig 2:**
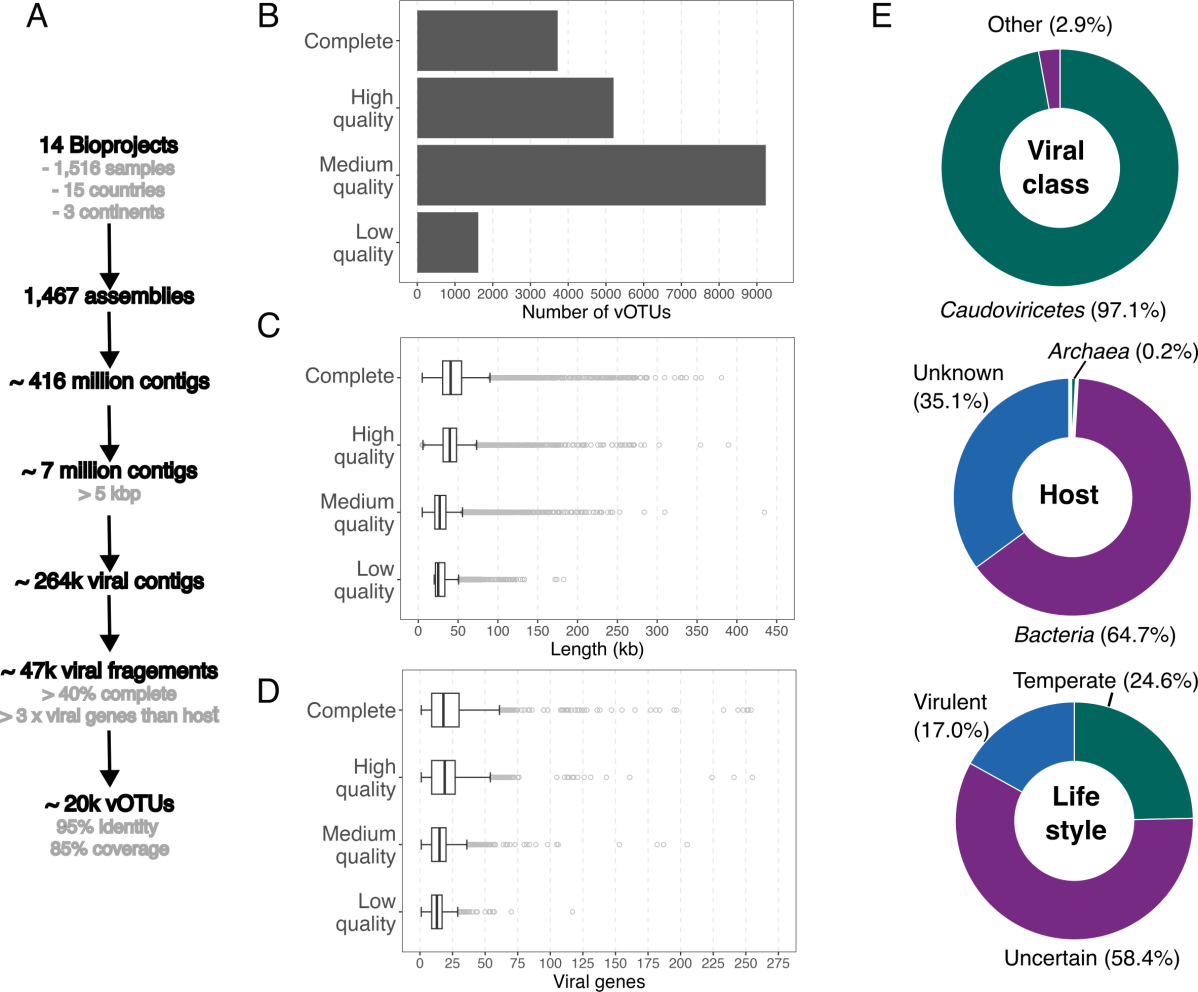
A global metagenomic analysis recovered 19,778 vOTUs from the chicken gut, predominantly *Caudoviricetes* associated with a bacterial host. (**A**) Overview of the assembly and vOTU recovery pipeline from 1,458 chicken gut metagenomes and 56 viral-enriched samples. (**B**) Number of vOTUs by genome quality. (**C**) Genome length distribution by genome quality. (**D**) Number of viral genes per vOTU across genome quality. (**E**) Proportion of vOTUs by viral class, predicted host domain, and lifestyle.

The selected viral genomes were clustered at the species level into viral operational taxonomic units (vOTUs) following the Minimum Information about an Uncultivated Virus Genome (MIUViG) recommended parameters of 95% average nucleotide identity (ANI) over 85% of the length of the shorter sequence (45). This analysis identified 19,778 vOTUs, of which 3,722 were complete, 5,202 high quality, 9,237 medium quality, and 1,617 low quality (Fig. 2B). Genome quality correlated positively with average genome length and a greater number of predicted viral genes (Fig. 2C and D).

From the total vOTUs, 18,902 were assembled from metagenomic samples (bulk-vOTUs), while only 876 were obtained from fecal viral-enriched samples (virus-like particle, VLP-vOTUs). Proportionally, more VLP-vOTUs (23.2%) were classified as complete genomes than bulk-vOTUs (18.6%). However, bulk-vOTUs were consistently longer than VLP-vOTUs (medians of 32.6 and 25.2 kbp, respectively), while maintaining a similar number of predicted viral genes (medians of 16 and 17 ORFs in bulk-vOTUs and VLP-vOTUs, respectively) (Fig. S1).

Using pairwise average amino acid identity (AAI) and gene-sharing metrics, we identify genus- and family-level vOTUs following the recommended analysis by Nayfach et al. (28). The 19,778 species-level vOTUs were grouped into 2,400 approximately genus-level clusters and 576 approximately family-level clusters. Accumulation curves demonstrated that while genus- and family-level viral diversity approaches saturation with increased sampling, species-level vOTU diversity continues to rise sharply, indicating a vast and still unsampled reservoir of species-level viral diversity in the chicken gut (Fig. S2).

Based on realm-level classification, we inferred the Baltimore classification for each species-level vOTU (46). Over 97.4% of the genomes were classified as double-stranded DNA (dsDNA) viruses, while approximately 2.5% and 0.3% were single-stranded DNA (ssDNA) and RNA viruses, respectively. The predominance of dsDNA vOTUs may reflect the bias of metagenomic sequencing approaches, which primarily recover dsDNA. Similarly, we can assume that RNA viruses are likely either retroviruses (true DNA intermediates) or represent misclassification artifacts resulting from incomplete assemblies or database limitations.

The majority of vOTUs were recovered from samples originating from China (42.1%), followed by Germany (12.1%), Poland (5.8%), Italy (5.6%), Bulgaria (5.2%), and other countries (29.2%) (Fig. S3). This distribution reflects the varying contributions of metagenomic data from each country, with 654 metagenomic samples originating from China, 560 from Germany, and 300 from other countries (Fig. 1A). Similarly, most vOTUs were recovered from feces samples (80.2%), followed by caeca (10.5%), ileum (4.8%), colorectum (1.7%), crop (1.2%), jejunum (0.7%), and duodenum (0.6%) (Fig. S4).

### Host prediction, taxonomy, and lifestyle

**Fig 3:**
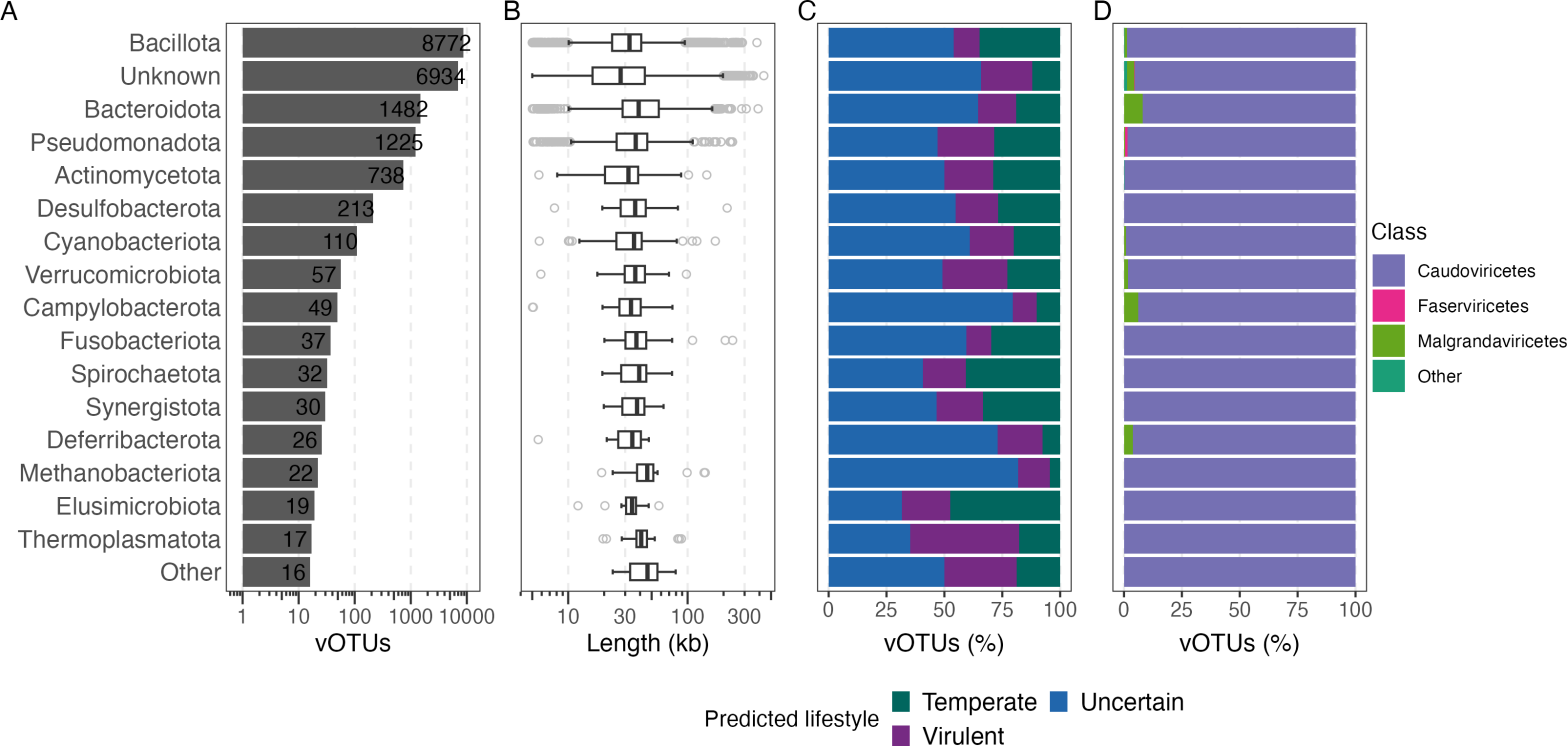
Chicken gut phages are largely associated with dominant chicken gut bacterial phyla like *Bacillota*, *Bacteroidota,* and *Pseudomonadota*. (**A**) Distribution of vOTUs across the predicted prokaryotic phyla host. The X-axis is depicted on a log scale. (**B**) Genome length distributions (in kb) of vOTUs associated with each phylum. The X-axis is depicted on a log scale. (**C**) Predicted lifestyle of vOTUs (temperate, virulent, or uncertain) stratified by host phylum. (**D**) Predicted viral class of vOTUs grouped per host phylum.

Identifying the bacterial hosts of phages is crucial for understanding their role in shaping microbiome composition, mediating horizontal gene transfer, and driving host–virus coevolution. Using an Integrated Phage Host Prediction tool (iPHoP) (47), we assigned hosts to the chicken gut viral genomes, identifying 12,796 bacteriophages, 49 archaeal viruses, and 6,934 viruses with no confidently predicted host (Fig. 2E). The archaeal viruses were predicted to infect five distinct archaeal phyla and 12 genera, while the bacteriophages were associated with 16 bacterial phyla and 624 genera (Fig. 3A). The majority of bacteriophages were associated with *Lactobacillus* (*n* = 1,539), *Limosilactobacillus* (*n* = 850), *Escherichia* (*n* = 718), *Bacteroides* (*n* = 623), *Ligilactobacillus* (*n* = 556)*,* and *Faecalibacterium* (*n* = 354), which are well-characterized genera of the chicken gut (Fig. S5) (48, 49).

Metagenomic studies have revealed a wide range of viral diversity that is not captured by current taxonomic ranks. Among the chicken gut vOTUs, 99.9% were classified at the class level, whereas only 3.8% could be assigned to an order and 7.4% to a family. In total, 12 viral classes were identified. *Caudoviricetes* was the dominant viral class, comprising 97.1% of all vOTUs, followed by *Malgrandaviricetes* (2.2%) and *Pisoniviricetes* (0.2%, Fig. 2E and Fig. 3D). *Caudoviricetes* and *Malgrandaviricetes* are double-stranded (dsDNA) and single-stranded (ssDNA) phages, respectively, that primarily infect bacteria. In contrast, *Pisoniviricetes* are RNA viruses. Among the identified viral families, we detected representatives of *Microviridae*, *Rountreeviridae*, *Autographiviridae*, *Drexlerviridae*, and *Herelleviridae*. In addition, 179 genomes were classified in the *Crassvirales* order, a group previously reported to be highly abundant in humans and other animals, including chickens (50, 51).

Furthermore, we used a bacteriophage lifestyle prediction tool (BACPHLIP) (52) to infer viral lifestyle, classifying the genomes as either temperate or virulent based on the presence of conserved protein domains associated with lysogeny. Virulent viruses are defined as obligately/strictly lytic, in contrast to temperate viruses capable of lysogeny. Approximately 4,876 genomes were classified as temperate and 3,353 as virulent (Fig. 2E and Fig. 3C). However, most genomes could not be classified into a specific lifestyle. Given that conserved proteins associated with temperate phages may be absent due to assembly limitations, we restricted BACPHLIP analysis to the complete genomes we had mined. Both approaches produced similar results, as 20.4% of the complete vOTUs were identified as temperate, 20.1% as virulent, and the rest as uncertain. The high proportion of unclassified genomes likely reflects the extensive novel viral diversity of chicken gut phages. This diversity is underrepresented in reference data sets used to train lifestyle prediction algorithms, highlighting a current limitation in accurately characterizing these newly identified viruses.

### Functional diversity of the chicken gut virome

**Fig 4:**
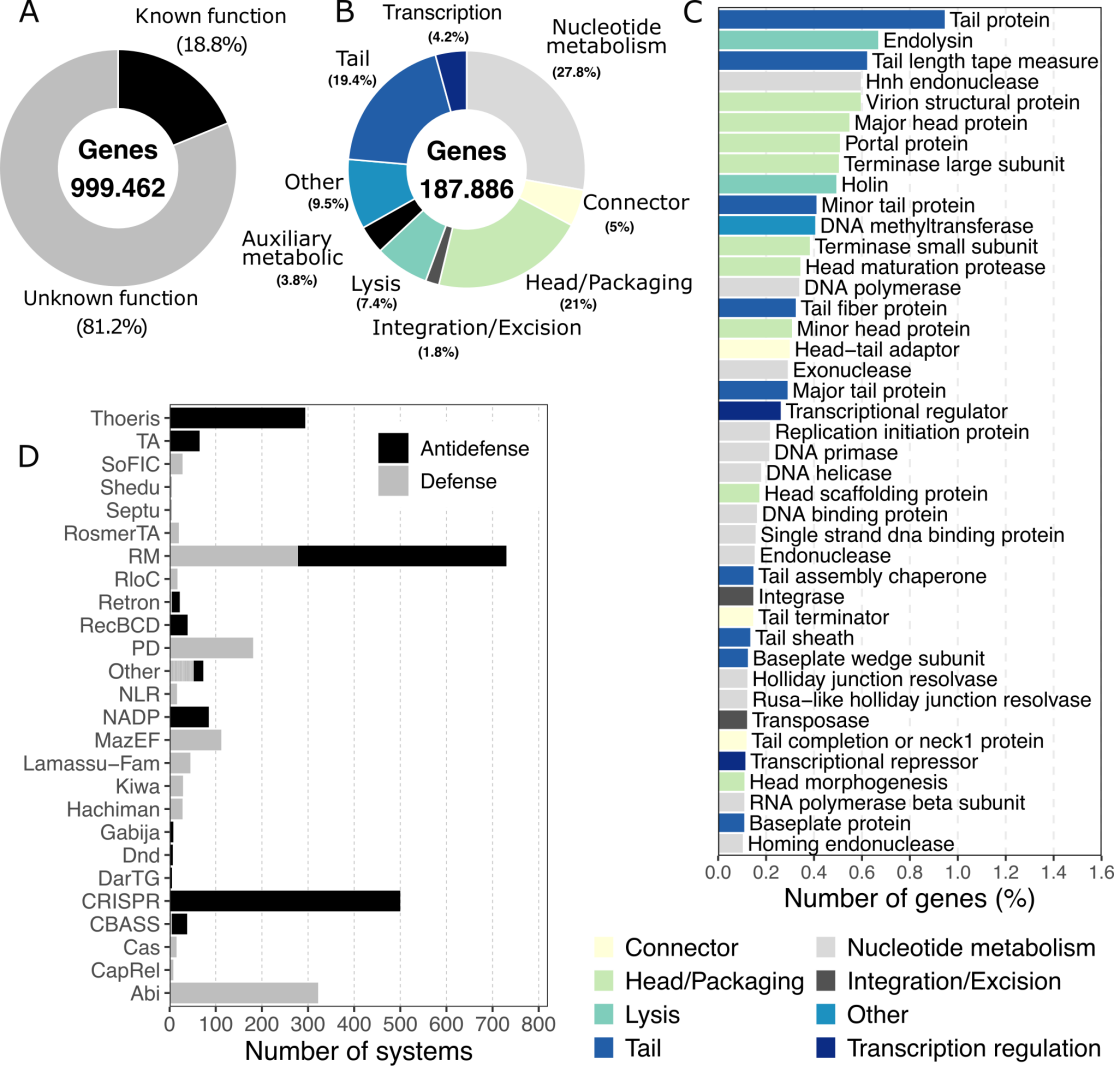
Chicken gut viral genomes harbor extensive functional novelty and defense and antidefense systems. (**A**) Proportion of the total genes with known and unknown function. (**B**) Proportion of genes with known function distributed between functional categories. (**C**) Functional annotation of the 40 most prevalent genes. (**D**) Defense and antidefense systems predicted in the viral genomes from the chicken gut.

To investigate the functional diversity of the chicken gut virome, we annotated viral genes using pharokka (53) and PHANOTATE (54), prediction tools optimized for bacteriophages and small proteins. In total, 999,462 protein-coding genes were predicted across the 19,778 viral genomes. Of these, only 187,886 genes showed homology to sequences in the Prokaryotic Virus Remote Homologous Groups database (PHROG), while the rest lacked assignment to any known biological function (Fig. 4A). This underscores not only the high degree of novelty within the chicken gut virome but also the largely uncharacterized functional potential of these viruses. Most of the annotated genes with assigned functions were associated with typical viral processes, including nucleotide metabolism, head and tail assembly, packaging, host cell lysis, and transcriptional regulation (Fig. 4B). Among these, the most prevalent categories were tail proteins, represented by 9,450 genes, followed by endolysins (6,676 genes), tail length tape measure proteins (6,214 genes), HnH endonucleases (5,948 genes), and virion structural proteins (5,947 genes) (Fig. 4C).

Approximately 4% of the protein-coding genes were identified as auxiliary metabolic genes (AMGs) and host takeover genes. The most abundant AMG was the phosphoadenosine phosphosulfate reductase, present in 578 vOTUs, which is an enzyme involved in the assimilatory sulfate reduction pathway (55). vOTUs carrying this enzyme were mainly associated with *Escherichia*, *Bacteroides,* and *Faecalibacterium*, but more than a third of the vOTUs could not be linked to a host. Although some AMGs involved in metabolic processes such as reductases, glycosyltransferases, and deacetylases were detected, most auxiliary genes were related to host defense systems against mobile genetic elements, including toxin-antitoxin systems, abortive infection systems, and restriction-modification systems.

The presence of defense and antidefense systems across viral genomes was surveyed using DefenseFinder (56), which detected 1,773 and 1,703 defense and antidefense genes across 2,280 vOTUs, respectively. The majority of defense systems were associated with abortive systems and restriction modification, while antidefense systems were associated with CRISPR and restriction modification (Fig. 4D). However, a wide diversity of system types was associated with both defense and antidefense (66 and 12, respectively), suggesting strong co-evolution between the chicken viruses and their respective hosts. The vOTUs harboring defense and antidefense systems were mainly associated with *Limosilactobacillus*, *Escherichia*, *Lactobacillus,* and *Ligilactobacillus*. Defense mechanisms are widespread across different environments, like the rumen (57), human gut, soil, and ocean (58).

### Viral diversity varies from bird to bird

**Fig 5:**
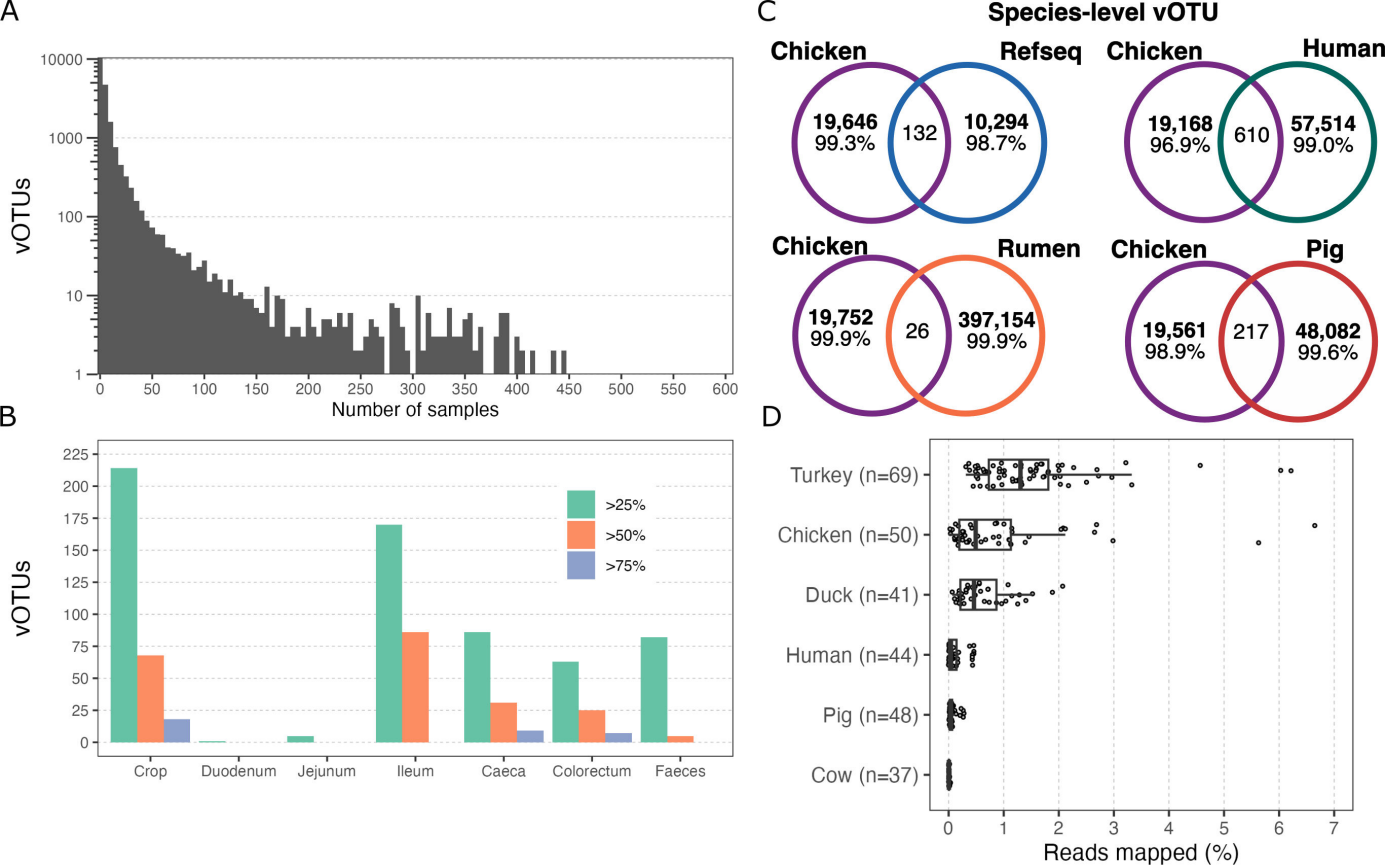
The chicken viral community is highly individualized and unique compared to other animal viromes. (**A**) Distribution of vOTUs across chicken gut samples. (**B**) Core vOTUs found in different fractions of samples, grouped by gut region. Each color represents the percentage of samples included in the calculation of the core virome. Core viromes were calculated in each gut section, excluding the rest of the samples. (**C**) Venn diagram showing the number of chicken gut vOTUs shared with Refseq viral, human gut (UHGV), rumen (RVD), and pig gut (PVD) collections of species-level vOTUs. (**D**) Prevalence of chicken gut vOTUs in other animal guts.

In chickens, multiple studies using amplicon sequencing and shotgun metagenomics have identified bacterial genera commonly found across individuals (4). On the other hand, these core members are not evident in viral communities. We observed that 10,487 vOTUs, accounting for 53% of the total, were present in only one or two samples (Fig. 5A), indicating that the chicken virome is highly individualized. Notably, no single vOTU was detected in at least 50% of the samples, and only 369 vOTUs were found in more than 100 samples, representing approximately 7% of all samples. Among the 10 most prevalent vOTUs, which were present in 435–607 samples, all belonged to the class *Caudoviricetes*. Host predictions revealed that only two of these vOTUs were associated with the bacterial genera *Metalachnospira* and *Anaerobutyricum*, while the hosts of the remaining vOTUs remain unidentified.

To identify unique vOTUs from the chicken gut, we clustered 19,778 vOTUs from our study with 10,419 viruses from RefSeq, 58,124 viruses from the human gut (UHGV), 397,180 viruses from several ruminants (RVD), and 48,299 viruses from the pig gut (PVD) at the species level. Surprisingly, we found that only 0.1%–3.1% of the chicken vOTUs clustered with the compared data sets (Fig. 5C), suggesting that the chicken gut harbors a largely unique and uncharacterized virome. This uniqueness likely reflects host-specific viral communities shaped by the distinct physiology, diet, and microbial composition of the chicken gut.

To further explore the host range and distribution of the chicken gut vOTUs, we assessed their read coverage across metagenomic samples from other animal hosts, including cows, pigs, humans, ducks, and turkeys. The highest proportion of recruited reads was observed in turkey, chicken, and duck samples, while read recruitment in human, pig, and cow samples was approximately 10-fold lower (Fig. 5D). These results suggest that bird-associated hosts may share a subset of vOTUs, potentially reflecting a conserved avian-specific prokaryotic and viral community.

### The composition of the chicken gut virome varies across different gut regions

To investigate whether viral communities also vary across gastrointestinal compartments, we grouped samples by gut region and calculated core members at various thresholds. Only a small fraction of vOTUs were consistently shared among samples within the same gut region (Fig. 5B). The highest numbers of shared vOTUs were detected in the ileum and crop, where more than 200 vOTUs occurred in at least 25% of samples, and a considerable proportion persisted across 50% or more of the samples. In contrast, the duodenum and jejunum harbored almost no vOTUs shared above the 25% threshold, highlighting their low overlap in viral community composition. The ceca, colorectum, and feces showed intermediate levels of shared vOTUs, but only a small subset reached prevalence in more than 75% of the samples. These results indicate that viral communities are highly individualized, with limited core membership across regions, and suggest that the ileum and crop represent the most stable reservoirs of recurrent vOTUs in the chicken gut.

**Fig 6:**
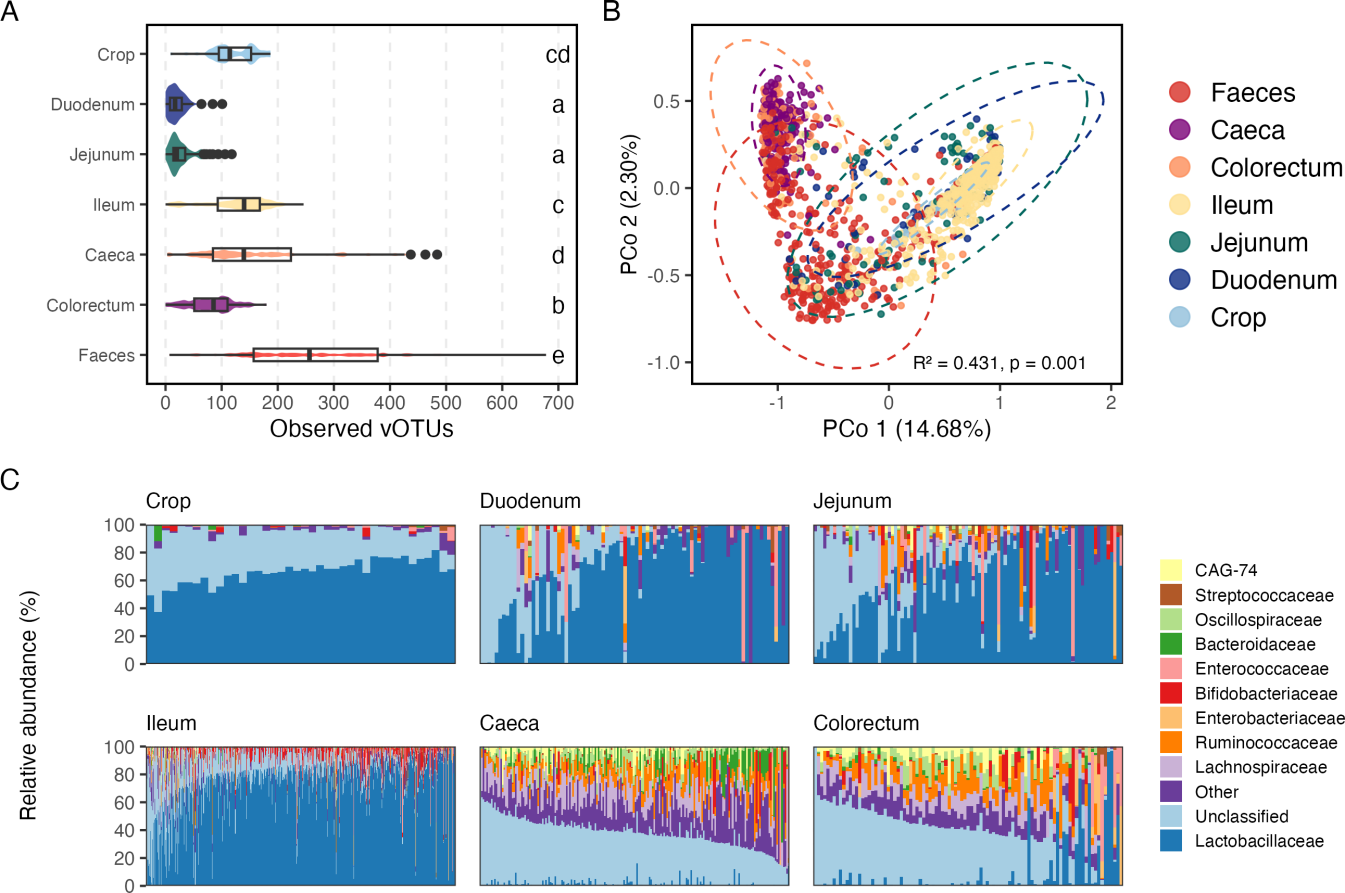
The chicken gut virome is structured along the intestinal tract with site-specific richness and composition. (**A**) Distribution of observed vOTUs in different gut regions. Letters indicate statistically significant differences between groups based on pairwise Wilcoxon rank-sum tests (*P* < 0.05). (**B**) Principal component analysis based on Bray-Curtis dissimilarities predicted by the abundance of vOTUs grouped by putative hosts at the family level. (**C**) Relative abundance of vOTUs grouped by their predicted host, at the genus level, across distinct gastrointestinal regions.

Given that prokaryotic diversity varies along different regions of the chicken gut, we investigated whether viral diversity follows a similar trend. We mapped raw reads against the mined vOTUs, using recruited reads as a proxy of the relative abundance of the total vOTUs across the chicken gut. Overall, the highest abundance of viruses was found in ileum samples (1.84% ± 1.15% mapped reads), followed by crop (1.64% ± 0.690%), caeca (1.62% ± 0.693%), jejunum (1.24% ± 2.59%), colorectum (1.19% ± 0.610%), and duodenum (1.10% ± 1.59%). Moreover, the number of observed vOTUs was significantly different between the different gut regions (Fig. 6A**,** Wilcoxon rank-sum test *P*<0.05). The highest average richness was observed in the feces and caeca samples, whereas the jejunum and ileum harbored significantly fewer vOTUs.

Principal coordinate analysis (PCoA) based on predicted putative hosts at the family level revealed a strong spatial stratification of phage communities along the chicken gastrointestinal tract (Fig. 6B, PERMANOVA *R*^2^ = 0.431, *P* = 0.001). Samples from the crop, duodenum, jejunum, and ileum formed a cluster, indicating that these upper gut regions harbor relatively similar phage–host patterns. In contrast, samples from the caeca, colorectum, and feces clustered separately. Feces showed a broader spread, reflecting higher heterogeneity. This structured distribution underscores that the chicken virome is not homogeneous but instead shaped by the physiological and microbial gradients that characterize the proximal and distal gut.

Also, vOTUs predicted to infect members of the *Lactobacillaceae* family were highly dominant in the proximal region of the chicken gut, including the crop (Wilcoxon rank-sum test *P* < 0.05), compared with the distal region. In contrast, vOTUs associated with *Lachnospiraceae*, *Ruminococcaceae*, and *Bacteroidaceae* were significantly more abundant in the distal region (Fig. 6C, Wilcoxon rank-sum test *P* < 0.05). These patterns mirror the niche specialization of bacterial communities in the chicken gut. The distribution of phages appears to be linked to the distribution of their bacterial hosts. This reinforces the idea that phage-host dynamics are major drivers of gut viral ecology and indicates co-evolution and adaptation of phages to their specific bacterial niches within the gut.

#### *Crassvirales* are low abundant and heterogeneous in the chicken gut

**Fig 7:**
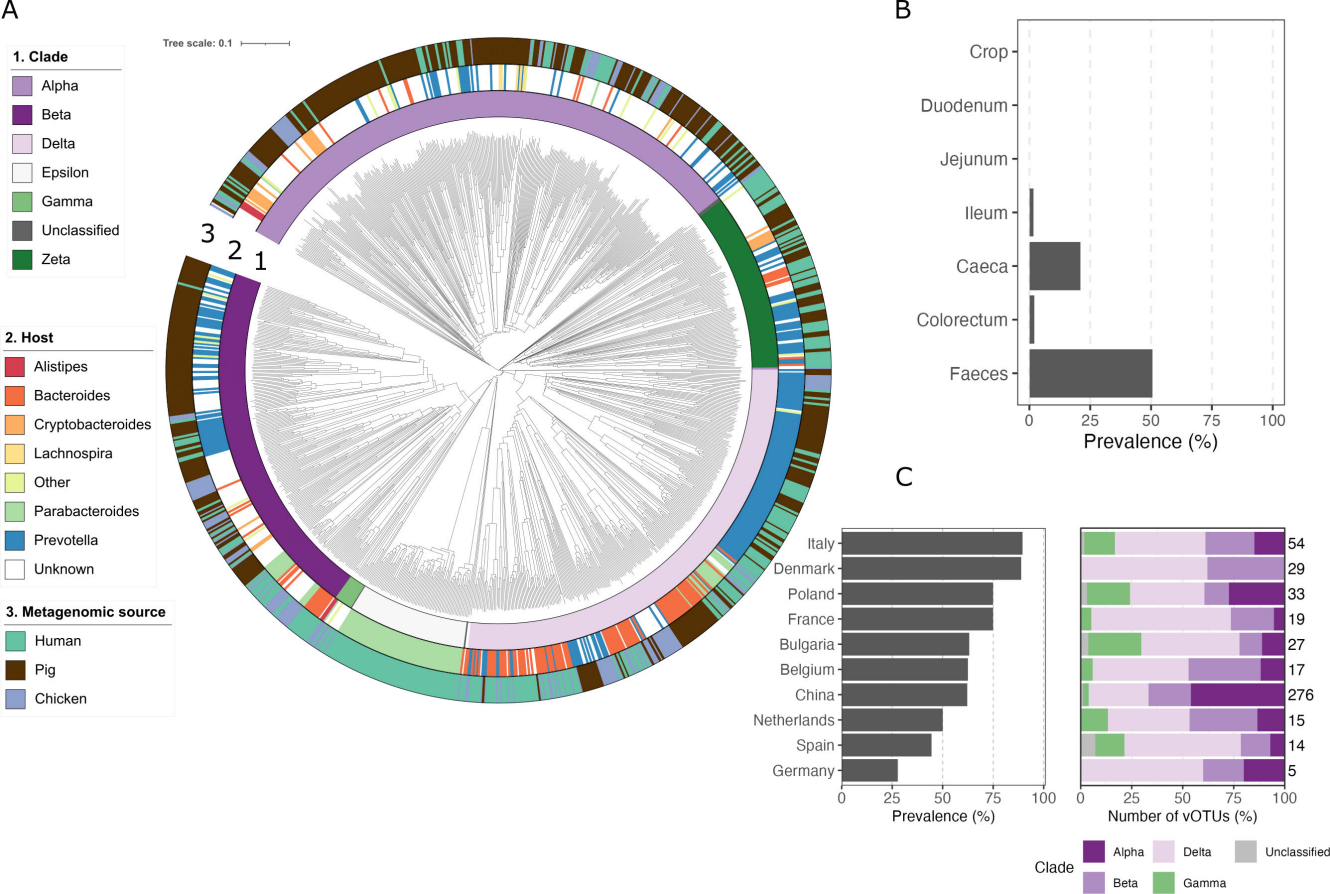
*Crassvirales* include both shared and host-associated lineages across humans, pigs, and chickens. (**A**) Proteomic tree of *Crassvirales* genome sequences based on genome-wide sequence similarities. Concentric annotation tracks indicate (1) *Crassvirales* clade assignment, (2) predicted bacterial host genus, and (3) the metagenomic sample source. (**B**) Prevalence of *Crassvirales* vOTUs along different regions of the chicken gastrointestinal tract. Positive prevalence was considered if at least one vOTU was detected in the sample. (**C**) Prevalence of *Crassvirales* vOTUs across different countries and the number of vOTUs grouped by clade. The number on the right represents the number of vOTUs detected across the samples of each country.

A total of 165 vOTUs members of the *Crassvirales* order (>50% complete genomes) were recovered from the chicken gut samples, with a median length of 97 kb and a maximum length of 105 kb. These vOTUs were assembled from feces (*n* = 152) and ceca samples (*n* = 13). Gene-sharing network analysis revealed that chicken *Crassvirales* vOTUs belong to the Alpha (*n* = 45), Beta (*n* = 46), Delta (*n* = 6), and Gamma (*n* = 64) clades, recently classified by the International Committee on Taxonomy of Viruses (ICTV) as *Intestiviridae*, *Steigviridae*, *Crevaviridae,* and *Suoliviridae* families (Fig. 7A; Table S6). Only four vOTUs could not be clustered with known families within the order *Crassvirales,* including the candidate clades Epsilon and Zeta, indicating the presence of potential novel families.

Analysis of *Crassvirales* prevalence across the chicken gastrointestinal tract revealed a marked localization in the distal gut. While *Crassvirales* were nearly absent in the upper region, they were detected in the caeca and reached their highest prevalence in feces (Fig. 7B). A positive prevalence was considered when at least one vOTU was detected per sample. To further describe their distribution across host populations, we compared the vOTUs prevalence in fecal samples from different countries collected in this study. Their prevalence varied by country, with the highest levels observed in some European countries like Italy and Denmark (Fig. 7C). Clade abundance also varied across countries, with Delta and Beta lineages consistently detected in all countries, whereas Gamma lineages showed a more restricted presence.

*Crassvirales* were initially described as highly prevalent and abundant in the human gut (59), and have since been detected in other animals, including primates and cats (60), pigs (61), and chickens (51). To assess whether chicken *Crassvirales* share similarities with those from other hosts, we compared our vOTUs against 335 and 526 *Crassvirales* vOTUs obtained from human and pig samples (61), respectively. To assess the overlap of *Crassvirales* diversity across hosts, vOTUs were clustered using vClust (62). At the species level, only one vOTU was shared between chickens and human vOTUs (Fig. S7). In contrast, genus-level clustering revealed greater shared diversity, suggesting that broader lineages may be conserved across different gut environments. Interestingly, among the genus-level vOTUs shared across chickens, humans, and pigs, one was identified as *Carjivirus*, a virus capable of alternating between phage and plasmid lifestyles across a broad host range (63).

Furthermore, we clustered the human, pig, and chicken *Crassvirales* genomes based on shared protein content and similarity using VIPTree (64). The resulting proteomic tree revealed a clade-based clustering of the analyzed genomes. Genomes recovered from the chicken gut clustered together with viral genomes derived from human and pig samples, indicating that phylogenetically related *Crassvirales* can occur across multiple host species (Fig. 7A). Approximately 81% of the analyzed genomes belonged to the Alpha, Beta, and Delta clades, each containing representatives from pigs, humans, and chickens. In contrast**,** *Crassvirales* from the Epsilon clade were derived exclusively from human samples, suggesting that while some *Crassvirales* lineages represent generalist gut-associated viral groups, others may be more host or context-restricted. Predicted bacterial hosts from all clades mainly belong to *Bacteroidota* and *Bacillota*, particularly the genera *Prevotella* and *Bacteroides*. The only exception was the Epsilon clade, which was exclusively associated with *Parabacteroides* (Fig. 7A). However, a substantial fraction of the chicken and pig-associated *Crassvirales* lacked confident host predictions, reflecting current limitations in host assignment for these viral lineages. Although *Crassvirales* were initially described as highly prevalent in the human gut, the detection of related lineages in pig, chicken, and other gut metagenomes supports the view that this viral order is broadly associated with vertebrate gut ecosystems rather than being restricted to a single host species.

### Viral richness and composition are associated with the geographical region, animal breed, and diet

**Fig 8:**
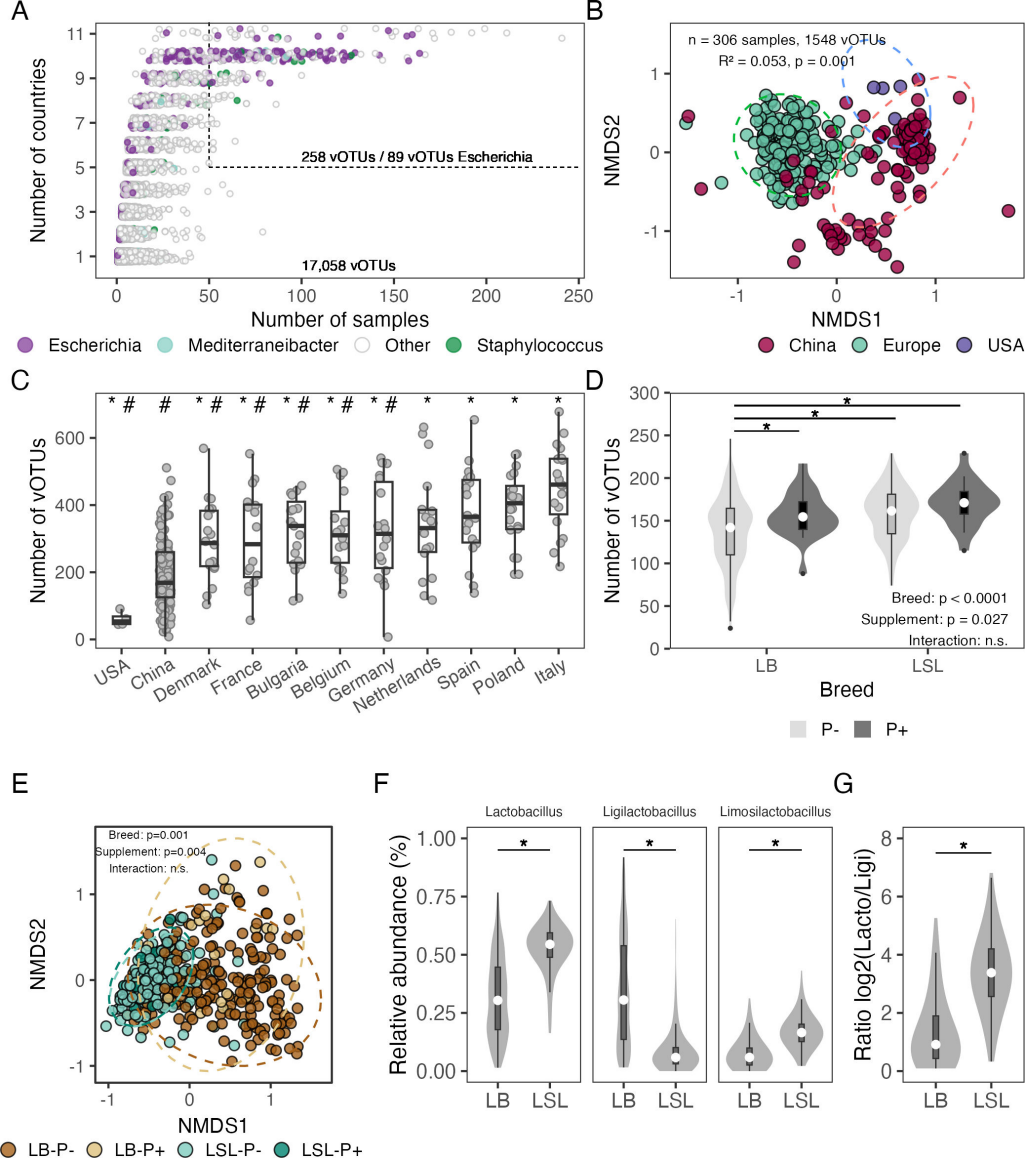
The geographical region, breed, and diet supplementation drive the chicken gut viral diversity and composition. (**A**) Frequency of vOTUs across fecal samples collected from several countries. Circles are colored based on the tree-top predicted bacterial host. Dashed line indicates vOTUs detected in > 50 samples and > 5 countries. (**B**) Non-metric multidimensional scaling (NMDS) of fecal samples based on Bray–Curtis dissimilarities of vOTU relative abundances, colored by geographic region. Only vOTUs present in 10 or more samples were used. (**C**) Number of vOTUs per fecal sample across countries. * indicates a significant difference between the country and Italy, while # indicates a significant difference between the country and China (Wilcoxon rank-sum test, *P* < 0.05). (**D**) Number of vOTUs in ileal samples stratified by chicken breed. Lohmann Brown Classic (LB) and Lohmann LSL-Classic (LSL) laying hens. * indicates significant pairwise contrasts of estimated marginal means (EMMs) on the response derived from a negative binomial generalized linear model (strain × diet; *P* < 0.05). n.s indicates non significant *P* > 0.05. (**E**) NMDS ordination of ileal samples grouped by breed and phosphorus supplementation. (**F**) Relative abundance of vOTUs associated with *Lactobacillus*, *Ligilactobacillus*, and *Limosilactobacillus* detected in ileal samples across chicken breeds. (**G**) Relative abundance ratio between vOTUs associated with *Lactobacillus/Ligilactobacillus* in ileal samples. * indicates a significant difference between the breeds (Kruskall-Wallis test, *P* < 0.05)

To investigate the ecological drivers of the chicken gut virome, we examined how viral diversity and composition vary across geographical regions, host breeds, and dietary supplementation. We first analyzed the frequency of vOTUs in fecal samples collected from multiple countries. Consistent with earlier observations, most vOTUs were detected in only a limited number of samples or countries (Fig. 8A). However, a small subset of vOTUs (258 vOTUs out of 17,058) showed broad prevalence, being detected in more than 50 fecal samples and across more than five countries. Notably, a substantial fraction of these widely prevalent vOTUs (89 vOTUs) were predicted to be associated with the bacteria *Escherichia*. Given the widespread occurrence of this bacterial genus in the chicken gut (1, 65), this pattern suggests that viruses linked to highly prevalent bacterial hosts may represent a stable and recurrent virome fraction of the chicken gut virome that persists across individuals and geographic regions, in contrast to the largely individualized virome.

At the community level, this balance between individualized and shared vOTUs was further reflected in the geographic distribution of the fecal samples (Fig. 8B). NMDS ordination based on Bray–Curtis dissimilarities of vOTU (present in more or 10 samples) relative abundances revealed partial clustering of samples from Europe, China, and the USA, indicating that viral community composition varies by region (PERMANOVA, *R*² = 0.053, *P* = 0.001), while still showing within region variability. These compositional differences were accompanied by significant differences in viral richness, with European samples having higher vOTU richness than those from China and the United States (Fig. 8C; Wilcoxon rank-sum test, *P* < 0.05). However, these geographic patterns should be interpreted with caution, as they may partly reflect differences in diet formulation, feeding regimens, breed, and management practices across studies rather than geographic location alone. Comparable results have been reported from the human gut virome between Chinese and non-Chinese individuals, where geographically distinct viral populations exhibit genetic differentiation and associations with host and microbiome-linked traits (66). Together, these results suggest that although individual-level variation dominates the chicken gut virome, regional factors are associated with shifts in viral diversity and composition.

To further explore host and management-related drivers of viral diversity, we focused on ileal samples for which detailed metadata were available. In this case, samples derived from two breeds, Lohmann Brown Classic (LB) and Lohmann LSL-Classic (LSL) laying hens, whose diets were supplemented or not with phosphorus. Viral richness was analyzed using a negative binomial generalized linear model with breed, diet, and their interaction as predictors. The vOTU richness differed significantly between breeds (Fig. 8D; breed *P* < 0.0001), suggesting that host genetic background can shape viral diversity in the ileum. Phosphorus supplementation was also associated with differences in viral richness (supplement *P* = 0.027), whereas no significant interaction between breed and supplementation was detected, indicating that dietary effects could occur independently of host genotype. These findings suggest that while diet can modulate viral diversity, breed effects remain the dominant driver. Consistent with these richness patterns, viral community composition in ileal samples was clustered primarily by breed, with a secondary effect of phosphorus supplementation (Fig. 8E, breed *P* = 0.001, supplement *P* = 0.004).

Finally, differences in ileal virome composition between breeds were also reflected in the predicted bacterial hosts of their vOTUs (Fig. 8F). Relative abundances of vOTUs linked to *Lactobacillus*, *Ligilactobacillus*, and *Limosilactobacillus* differed significantly between breeds (Fig. 8G). LB chickens seem to have a more balanced community between *Lactobacillus* and *Ligilactobacillus* phages, while LSL chickens are dominated by *Lactobacillus* phages. Together, these results support the idea that the structure of the chicken gut virome is closely coupled to the underlying bacterial ecosystem and is shaped by the combined effects of host genetics, diet, and broader environmental context.

## DISCUSSION

Recent culture-dependent and culture-independent studies have demonstrated that viral communities exert significant influence on prokaryotic populations, affecting their composition, function, and evolutionary dynamics. Consequently, mining viral sequences from existing metagenomic data sets has emerged as an area of growing interest to elucidate the role of viruses within microbial ecosystems. By combining large-scale data mining of publicly available metagenomic samples and the extraction of metagenomic DNA from our own samples, we identified thousands of viral sequences from the chicken gut. We assembled 47,092 medium to high-quality viral genomes representing 19,778 species-level vOTUs. The collected data set reveals extensive and previously uncharacterized viral diversity that is highly individualized, largely absent from existing catalogs of reference genomes, and distinct from the viral community profiles of other animals (28, 29). This diversity is evident not only in the large number of newly identified species-level vOTUs but also in the high proportion of uncharacterized protein-coding genes, which account for nearly 80% of the total data set. Similarly, prior metagenomic studies of the chicken gut have reported numerous novel bacterial species and genera, alongside a considerable proportion of genes with unknown or database-unmatched functions (67). These findings underscore the substantial unexplored microbial and viral genetic diversity in the chicken gut, highlighting the need for further functional and ecological investigation.

Linking host-virus relationships is essential as phages can influence bacterial population densities, gene transfer, and metabolic processes, potentially affecting gut health, immunity, and nutrient absorption. Using several alignment-based and alignment-free methods, either phage-based or host-based (47), we predicted the host of the collected viral genomes. Chicken viral genomes are associated with the core members of the chicken gut, which are known to play essential roles in the digestion of complex carbohydrates and fiber, production of SCFA, maintaining gut pH, inhibiting pathogenic bacteria, and maintaining gut health overall (68). Additionally, those vOTUs linked with the core microbiome carry auxiliary metabolic genes that can modulate host metabolism. For example, the detection of phage-encoded assimilatory sulfate reduction genes in chicken gut viruses suggests that viral communities may modulate host sulfur metabolism, potentially enhancing cysteine and methionine biosynthesis and influencing microbial redox balance in the intestine (69). Although such genes have also been identified in human-associated phages (70), they appear to be inactive, underscoring the need to further investigate their functional roles and expression in the chicken gut ecosystem. Moreover, robust classification of PAPS reductase as an AMG in our data set requires additional analyses, such as examining its synteny with viral hallmark genes and calculating the absence of adjacent bacterial host genes.

Although the gut is compartmentalized and distinct microbial communities are known to inhabit each region (71, 72), most studies on the gut virome rely exclusively on fecal samples. Hence, it remains unclear to what extent viral communities reflect this spatial structuring (73, 74). Our results demonstrate clear proximal–distal differences, confirming that viral ecology is also strongly shaped by gut compartmentalization. This indicates that fecal samples capture only a subset of the viral diversity, primarily reflecting the distal gut, and thus cannot fully resolve the ecological dynamics of phage–host interactions across the entire gastrointestinal system. By integrating viral richness, community composition, and host predictions, our study demonstrates that the virome mirrors the functional compartmentalization of the gastrointestinal tract. This pattern is driven by the dominance of *Lactobacillaceae* members in the proximal regions, where their close association with the mucosa (75) and adaptation to bile acids (76, 77) may promote dense host populations that support abundant phage communities. However, distal regions are structured by more diverse bacterial communities, indicating that different host groups contribute to compartmentalization along the gut.

Because viral richness, community composition, and host availability vary across the chicken gut, phage life strategies are likely to shift accordingly. In the proximal gut, where *Lactobacillaceae* populations can be dense and stably associated with the mucosa, phages might adopt more temperate, lysogenic strategies (25, 78). In contrast, distal gut regions, where bacterial densities fluctuate, might favor lytic dynamics that constrain the dominance of certain bacterial taxa (79). This shift in phage and host dynamics across gut compartments could contribute to the variety of viral niches we observed, reinforcing the notion that fecal samples cannot fully capture the complexity of *in situ* viral dynamics.

Our analysis also provides new insights into the ecology of members of the *Crassvirales* order within the chicken gut, highlighting their variation across hosts and geographical regions. *Crassvirales* are among the most abundant phages in the human gut microbiome (51, 59, 80), yet their diversity and distribution in non-human hosts remain less explored (51, 60, 61). *Crassvirales* have been previously found in chicken viromes, but cross-contamination of human fecal material could not be ruled out due to their heterogeneous prevalence (51). By resolving their occurrence across six regions of the chicken gastrointestinal tract, we showed that *Crassvirales* are primarily restricted to the distal gut, with the highest prevalence in the caeca and feces. *Crassvirales* are not largely shared between humans, chickens, and pigs, but representatives are distributed across multiple phylogenetic clades, except for the clades Epsilon and Zeta (81). This indicates that although the specific lineages vary among host species, the broader *Crassvirales* radiation is widespread across animal gut ecosystems. Despite their phylogenetic divergence, these phages appear to converge on similar bacterial hosts, predominantly within the *Bacteroidota phylum*, including *Bacteroides*, *Prevotella,* and *Parabacteroides* (61, 82, 83). Such patterns suggest that host availability could mainly drive their persistence across diverse animal guts.

Geographic differences in viral composition and richness further suggest that regional production practices, diet formulation, or environmental exposures could be associated with systematic shifts in the chicken gut virome. Although individual variability remains high, the differences observed between geographical regions imply that the virome responds to population-level factors that also could influence growth performance, feed efficiency, and disease risk (84). Similar geographical structuring reported in the human gut virome supports the idea that viral communities, like bacterial ones, are shaped by broader ecological contexts, with potential consequences for host physiology (66).

Additionally, the predominance of rare vOTUs across samples indicates strong host and environmental filtering of viral communities. However, the presence of a small subset of widely prevalent vOTUs, particularly those associated with *Escherichia*, implies that globally prevalent bacterial hosts may drive a conserved viral core across geographical regions. This pattern underlines the interaction between local ecological structuring and host-driven viral selection in shaping the gut virome, as observed in humans (85). Similarly, analysis of the Unified Human Gastrointestinal Virome (UHGV) showed the presence of a small fraction of hyperprevalent vOTUs (detected in ≥50 samples and ≥3 countries) across human populations (86). Interestingly, those hyperprevalent vOTUs tend to harbor genetic factors that enable a broad host range. Together, these observations suggest that gut viral prevalence across geographical regions is shaped by a combination of host availability and phage-specific traits, rather than by bacterial host abundance alone.

Also, the association between virome diversity and chicken breed underscores the role of host genetic background in shaping viral communities. Differences in viral richness and composition between different breeds suggest that host factors influencing gut morphology, immune function, or bacterial community composition also could indirectly affect viral ecology. Similar patterns have been reported in cattle, where animals sharing breed and feeding regimens have greater overlap in gut vOTUs compared with animals differing in breed and diet (29). From a production point of view, this is particularly important because breed selection is already a central component of poultry breeding programs, and virome composition may contribute to variability in nutrient absorption, resilience to stress, and susceptibility to dysbiosis, as observed in humans and other animals (87, 88).

Even though our data set has revealed a broad diversity of novel elements, it is far from complete. Recently, Ji-Xin Zhao et al. (89) and Yanan Wang et al. (1) published chicken gastrointestinal virome collections derived from thousands of chicken gut metagenomic data sets. Similar to our collection, these catalogs consist of medium- to full-length viral genomes, clustered into non-redundant vOTUs that primarily represent novel viral diversity compared to other viral compendia. However, after clustering both collections into species-level vOTUs (95% average ANI over 85% of the length of the shorter sequence), we identified only 7,552 shared vOTUs. Consequently, our data set uniquely contributes 12,226 novel vOTUs, and together, both collections represent 51,606 species-level vOTUs. These differences may be attributed to methodological differences, such as assembly strategies, viral prediction approaches, and quality-filtering procedures. For example, we also tested VirSorter2 in combination with geNomad to annotate viral contigs, but this did not increase the number of recovered vOTUs. Because geNomad provides higher overall classification performance across all sequence length ranges, only viral contigs with >40% estimated completeness predicted by geNomad were retained for this study. Integrating both collections, along with future data sets, provides a valuable resource for developing a unified chicken gut virome, similar to recent efforts for human gut viral catalogs (https://github.com/snayfach/UHGV). Such a unified virome database could facilitate the identification and taxonomic annotation of viruses in metagenomic samples derived from the chicken gut.

## MATERIALS AND METHODS

### DNA extraction and sequencing of chicken gut metagenomic samples

For this study, a total of 539 chicken gut samples were obtained from two independent experiments conducted at Hohenheim University. The studies were approved by the Regierungspräsidium Tübingen (HOH50/17 TE and HOH67-21 TE) and conducted in accordance with animal welfare regulations. Animals were housed at the Hohenheim Agricultural Experimental Station (Unterer Lindenhof, Eningen, Germany). In the first study, metagenomic DNA was extracted from the crop (*n* = 40), ileum (*n* = 24), and caeca (*n* = 40) of LB and LSL laying hens (4). In the second study, 220 LB and 220 LSL, representing distinct genetic backgrounds, were raised in floor pens on deep litter bedding based on the standard procedure of the experiment station until high laying performance was achieved (90). For both experiments, DNA from the intestinal content was extracted using the commercial FastDNA SPIN Kit for Soil (MP Biomedicals LLC, Solon, OH) following the manufacturer’s instructions and adjusting the initial sample amount to 250 mg as previously described (91). All DNA samples were stored at −20°C until sequencing. DNA extractions were quality controlled by measuring the concentration and purity with a Spectrophotometer NanoDrop 2000 (Thermo Fisher Scientific, Waltham, MA, United States). Additionally, total DNA was quantified using the QuantiFluor dsDNA System kit following the manufacturer’s instructions (Promega, Heilbronn, Germany) in a Qubit 4 Fluorometer (Thermo Fisher Scientific, Darmstadt, Germany). DNA extractions yielding >20 ng were sequenced on the Illumina NovaSeq 6000 platform by Novogene Company Ltd. (Cambridge, UK). All the sample sequences were deposited in the European Nucleotide Archive (ENA) under the bioprojects PRJEB60928 and PRJEB84467.

### Collection of public chicken metagenomic samples

To expand the chicken gut data set with samples from different countries and gastrointestinal regions, 974 chicken gut DNA metagenomic samples were collected from public databases using the SRA-toolkit v3.1.1 (https://github.com/ncbi/sra-tools). From each sample, metadata were downloaded from the NCBI Biosample database (92). The collected metagenomic samples comprised approximately 3.1 TB of data, corresponding to 2.4 × 10^10^ reads and 3.5 × 10^12^ base pairs. These metagenomic samples included samples from Belgium (*n* = 16), Bulgaria (*n* = 19), Denmark (*n* = 18), France (*n* = 16), Germany (*n* = 18), Italy (*n* = 19), the Netherlands (*n* = 18), Poland (*n* = 20), and Spain (*n* = 18), all from project PRJEB22062. Additional samples were from Switzerland (*n* = 52, PRJNA802076), the United States (*n* = 33, PRJNA375762, PRJNA601052, and PRJNA977671), Canada (*n* = 37, PRJNA666163), China (*n* = 635, PRJNA340908, PRJNA408020, and PRJNA417359), India (*n* = 5, PRJEB9171), and Malaysia (*n* = 4, PRJNA291299). Samples were obtained from various sections of the chicken gut, including the crop (*n* = 40), duodenum (*n* = 99), jejunum (*n* = 99), ileum (*n* = 558), caeca (*n* = 214), colorectum (*n* = 99), and feces (*n* = 404). Details of the sample collection are provided in Table S1.

### Read cleaning, contigs assembly, and filtering

Quality control of the raw reads was performed using Trim Galore v0.6 with the auto detection and --paired options (https://github.com/FelixKrueger/TrimGalore), and host/feed DNA was removed by Bowtie2 v2.5.3 (93) using the chicken (GCA_024206055.2) and feed (GCA_902167145.1) reference genomes. After, read quality was evaluated using fast-qc v0.12.1 (https://github.com/s-andrews/FastQC). Assemblies were created for each sample with MegaHit v1.2.9 with the --presets and --meta-sensitive options (43). Due to low assembly quality, 47 samples were discarded. Assembled contigs were filtered based on length with a threshold of 5,000 bp using BBtools v37.62 (https://sourceforge.net/projects/bbmap/).

### Phage detection and genome quality evaluation

To identify and annotate viral contigs longer than 5,000 bp, we applied the geNomad v1.8 (94) end-to-end pipeline across the filtered 1,467 assemblies. Post-classification filters were applied using the --conservative presets. Briefly, the presets included min-score = 0.80, mad-fdr = 0.05, min-number-genes = 1, and min-virus-hallmarks = 1. After, CheckV v1.0.3 (44) and the end-to-end command were used to remove host contamination, estimate genome completeness, predict closed genomes, and summarize the quality of all the contigs identified as viral. Viral sequences were retained if all the following conditions were met: completeness ≥ 50, viral genes > 0, and viral genes > 3× than the host genes. Also, low-quality sequences were kept if all the following conditions were met: contig length ≥ 20,000, completeness ≥ 40, viral genes > 0, and viral genes > 3× than the host genes. Filtering parameters were tuned following the recommendations in the Viral sequence identification SOP with VirSorter2 V.2 (95).

### Dereplication, taxonomy, and host prediction

Filtered viral sequences were clustered based on pairwise Average Nucleotide Identity (ANI) following the CheckV supporting code (https://bitbucket.org/berkeleylab/checkv/src/master/). Briefly, Blast+ v2.13.0+ and makeblastdb were used to create a database including all the viral sequences (96). Next, an all-vs-all blastn –max_target_seqs 10,000 was performed using the sequences and the created database. After, ANI was calculated by combining the local alignments between sequence pairs. Lastly, a CD-HIT-like clustering was performed using the Minimum Information about an Uncultivated Virus Genome (MIUVIG) recommended parameters (--min_ani 95 --min_tcov 85 --min_qcov 0) (45). Genus-level and family-level vOTUs were clustered based on Amino Acid Identity (AAI) following the supplementary code provided in https://github.com/snayfach/MGV. Briefly, protein-coding genes were predicted from each viral genome using pharokka v1.7.2 (53), and all-versus-all protein comparisons were performed using DIAMOND BLASTP (*E*-value <1e-5, ≥50% query and subject coverage). AAI between genome pairs was calculated as the average percent identity across all shared protein hits. To construct genus- and family-level clusters, edges were filtered based on AAI and gene-sharing thresholds: genus-level clustering required ≥20% shared genes and ≥40% AAI, while family-level clustering used a relaxed threshold of ≥10% shared genes and ≥20% AAI. The resulting pairwise similarity matrices were clustered using the Markov Clustering Algorithm (MCL) with inflation parameters of 2.0 (genus) and 1.2 (family).

The vOTUs accumulation curves were calculated using the function specaccum (method = “random”, permutations = 1,000) from the “vegan” R package (97). Taxonomic assignments of the dereplicated sequence were obtained using the annotate module of genNomad v1.8. The geNomad virus taxonomy is based on the ICTV’s VMR lineages. Phage host was predicted using iPHop v1.3.3 (47) and a collection of host genomes extracted from GTDB, IMG, and MGnify MAG collections. IPHop is built upon RaFAH (98), WIsH (99), PHP (100), and VirHostMatcher (101).

### Lifestyle and functional prediction

The lifestyle of the vOTUs, whether temperate, lytic, or uncertain, was determined from conserved protein domains using BACPHLIP v0.9.3 (52). Additionally, BACPHLIP was tested using only complete genomes, as incomplete genome assemblies can lack the integrases often occurring at the ends of prophage genomes. Both approaches yielded similar results. Viral genes were predicted using pharokka (53) and PHANOTATE v1.5.1 (54), which are gene prediction tools tailored explicitly to bacteriophages and small genes. Afterward, functional annotations were assigned by matching each predicted coding sequence (CDS) to the PHROGs, CARD, and VFDB databases using MMseqs2 v13.45,111 (102). Lastly, anti-phage defense systems and antidefense proteins were searched across all viral genomes using DefenseFinder v1.3.0 (56) and its models based on MacSyFinder architecture (103).

### Clustering and comparison of *Crassvirales*

In total, 336 and 525 *Crassvirales* vOTUs obtained from human and pig (61) samples were compared against 165 *Crassvirales* vOTUs mined from our chicken samples. vOTUs were clustered using gene-sharing networks and classified as members of the Alpha, Beta, Delta, and Gamma clades using vContact3 (104). Human *Crassvirales*, previously identified, were used as a reference to define the candidate clades Epsilon and Zeta. vOTUs were clustered at species- and genus-level, performing VIRIDIC-like analysis by calculating the total ANI (tANI) between virus genomes and classifying these viruses into species and genera based on 95% and 70% tANI, respectively (62). *Crassvirales* putative hosts were predicted using iPHoP as previously described; only the predicted highest score was used. The proteomic tree was generated using VIPTree (64) and the *Crassvirales* genome sequences. The tree is based on genome-wide sequence similarities computed by tBLASTx. The proteomic tree was visualized and annotated using iTOL (https://itol.embl.de).

### Metagenomic read recruitment and comparison with other viral data sets

Read mapping was performed on viral genomes to calculate their abundance and read coverage using two different sets of samples. First, all clean reads from the different chicken gut regions used in this study were mapped to the viral genomes. Second, gut metagenomic samples from chicken (*n* = 50, PRJEB22062), turkey (*n* = 69, PRJEB39685), duck (*n* = 41, PRJNA1064443), human (*n* = 44, PRJEB17632, PRJNA421881, and PRJNA473126), pig (*n* = 48, PRJEB22062), and rumen cow (*n* = 37, PRJEB34458) were collected and mapped to the viral genomes. None of the second set of samples was used to create the collection. Newly collected metagenomic reads were cleaned and quality-controlled as previously described. Also, host contamination was removed using Bowtie2 and their respective genomes, turkey (GCA_000146605.4), duck (GCA_015476345.1), human (GRCh38), pig (GCA_000003025.6), and cow (GCA_002263795.4). Briefly, read mapping was done using coverM (105) following previously recommended thresholds for the assessments of viral community composition, --min-read-percent-identity 90 --min-read-aligned-percent 75 --min-covered-fraction 75 (106). Additionally, to identify the novelty of the chicken viruses, all viral genomes were clustered into species-level vOTUs based on 95% ANI and 85% alignment fraction with three different data sets as previously described. The three datasets were 10,419 viruses from RefSeq obtained using INPHARED (107), 54,118 viruses from the human gut (28), 397,180 viruses from several ruminants (29), and 48,299 viruses from the pig gut (108).

### Data wrangling and statistics

All data wrangling and statistical analyses were performed in R v4.1.2 (109), and the packages “tidyverse” (110), “data.table”, “vegan”, “MASS”, “pheatmap”, “ggdist”, “patchwork”, and “ggtext” were used. Median differences between the number of vOTUs and shared vOTUs were compared using the Kruskal–Wallis test and the function kruskal.test() in R. Bray–Curtis dissimilarities of vOTU relative abundances and vOTUs grouped by host were calculated using the vegan package. Permutational Multivariate Analysis of Variance (PERMANOVA) with the function adonis2() was used to test whether community composition differs among groups. Viral richness between breeds was compared using a negative binomial generalized linear model with breed, diet, and their interaction as predictors and the function glm.nb() from the MASS package.

## Data Availability

The collection of 47,092 viral genomes, the 19,778 dereplicated species-level vOTUs, and File S2 can be found in Zenodo, https://zenodo.org/records/15063554. Metagenomic samples used in this study are publicly available and can be downloaded using the provided Bioproject IDs in the supplementary materials. Workflows to analyze the data can be found as JupyterLab notebooks in https://github.com/SebasSaenz/chickenphages, as well as other R scripts for data wrangling and plotting.

## SUPPLEMENTAL MATERIAL

Supplemental figures msystems.00191-26-s0001.docx Figures S1 to S7.

Supplemental tables msystems.00191-26-s0002.xlsx Tables S1 to S6.

ASM does not own the copyrights to Supplemental Material that may be linked to, or accessed through, an article. The authors have granted ASM a non-exclusive, world-wide license to publish the Supplemental Material files. Please contact the corresponding author directly for reuse.
