# Supplementary material for: The chicken gut virome: spatial structuring and extensive diversity of 19,778 viral populations
Source: mSystems. 2026 Mar 30;11(4):e00191-26. doi: 10.1128/msystems.00191-26 (PMC13098223; doi:10.1128/msystems.00191-26)
Supplement: Supplemental figures — Figures S1 to S7. [file msystems.00191-26-s0001.docx]

**SUPPLEMENTARY MATERIAL**

**A metagenomic collection of 19,778 viruses reveals the diverse virome of the chicken gut**

**Johan S. Sáenz^1,2^, Timur Yergaliyev^1,2^, Bibiana Rios-Galicia^1,2^, Jana Seifert^1,2^ & Amelia Camarinha-Silva^1,2^**

^1^Institute of Animal Science, University of Hohenheim, Emil-Wolff-Str. 6-10, 70599 Stuttgart, Germany

^2^HoLMiR—Hohenheim Center for Livestock Microbiome Research, University of Hohenheim, Leonore-Blosser-Reisen Weg 3, 70599 Stuttgart, Germany

**
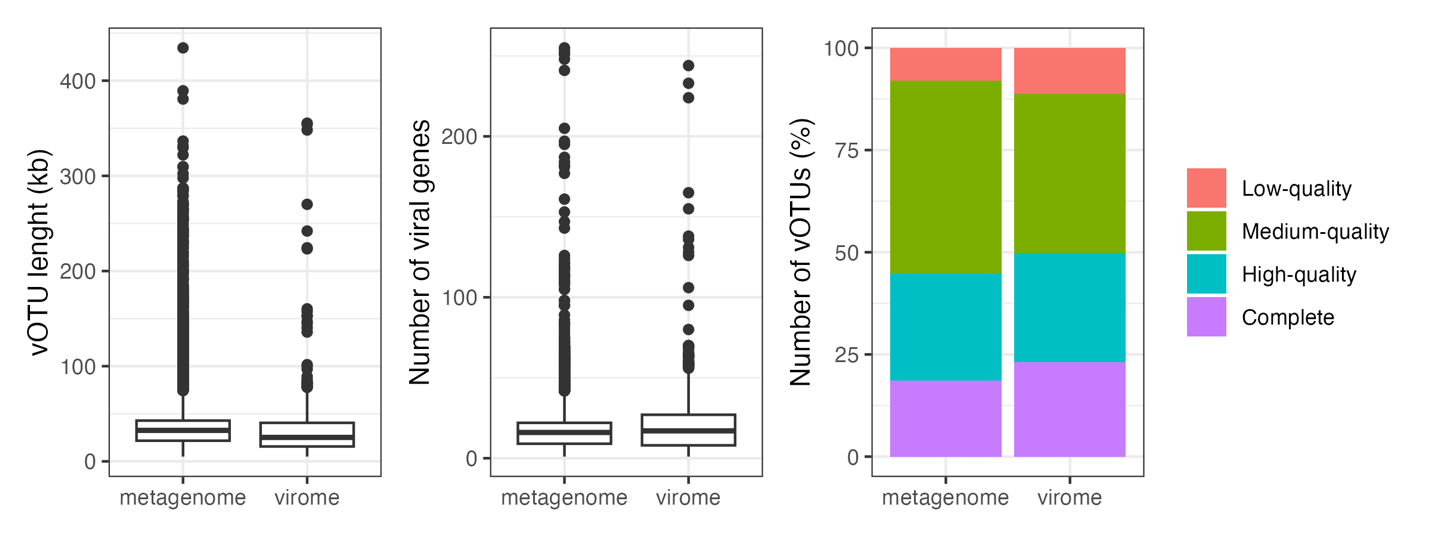
**

**Figure S1. Comparison of viral operational taxonomic units (vOTUs) recovered from metagenomes and viral-enriched samples.** A) Distribution of vOTU genome lengths (kb). B) Distribution of the number of predicted viral genes per vOTU. C) Quality categories of vOTUs based on genome completeness and contamination estimates.


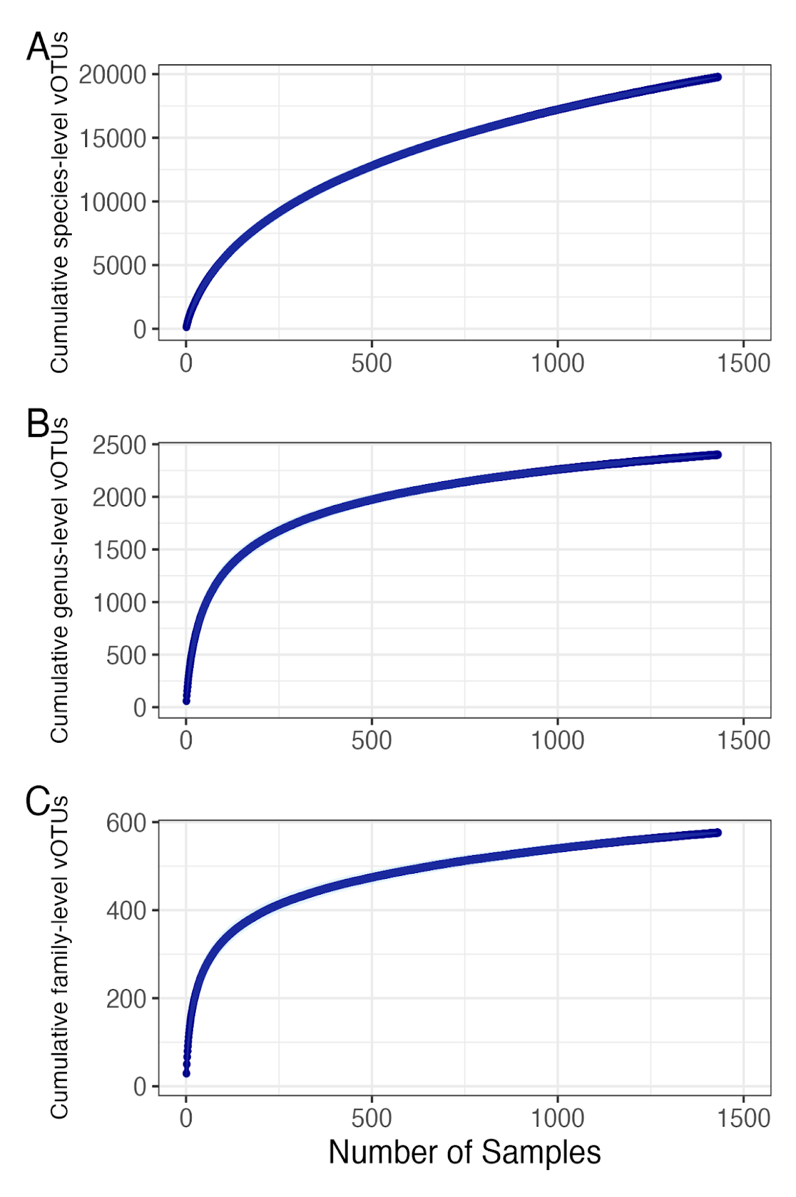


**Figure S2. Saturation curves of viral diversity across taxonomic levels.** Accumulation curves show the cumulative number of viral operational taxonomic units (vOTUs) detected at the species (A), genus (B), and family (C) levels as a function of the number of metagenomic samples analyzed.


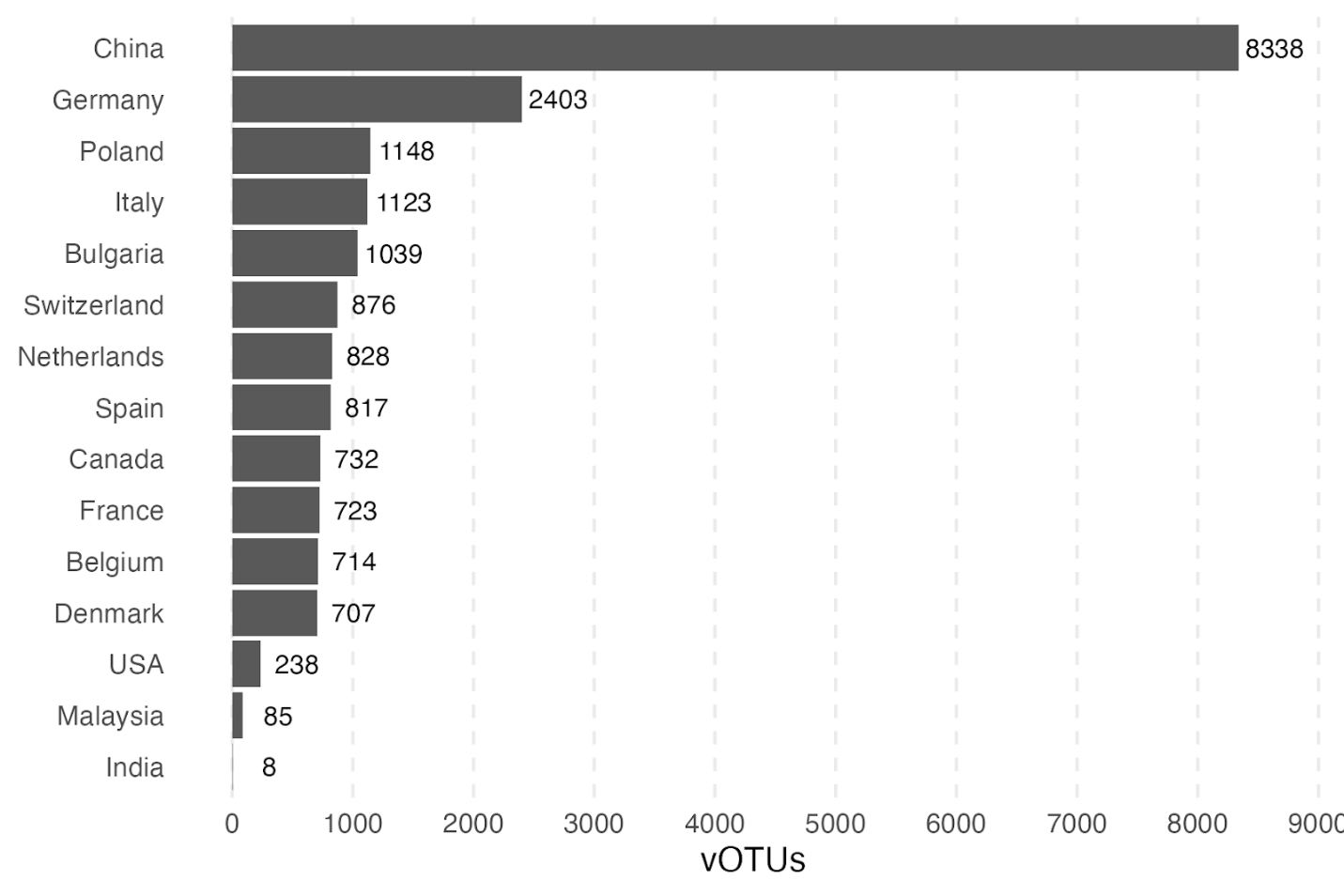
**Figure S3. Number of species-level vOTUs (n=19778) mined per country of sample origin.**

Samples were obtained from Belgium (n = 20), Bulgaria (n = 19), Canada (n = 40), China (n = 654), Denmark (n = 20), France (n = 20), Germany (n = 560), India (n = 5), Italy (n = 20), Malaysia (n = 4), the Netherlands (n = 20), Poland (n = 20), Spain (n = 20), Switzerland (n = 56), and the USA (n = 36), totaling 1,514 samples across all countries.


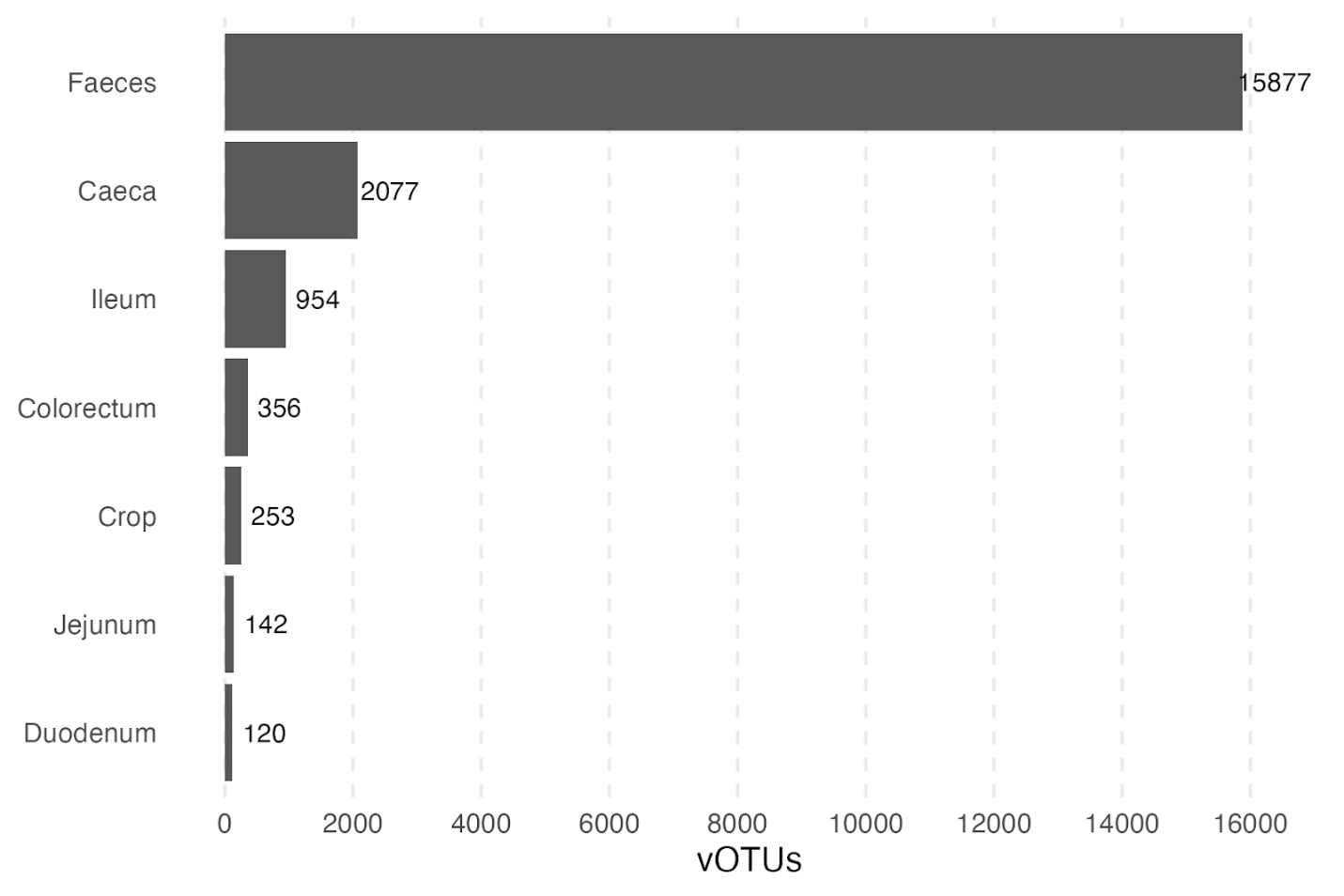


**Figure S4. Number of species-level vOTUs (n=19778) mined per gastrointestinal region.** Samples were distributed across faeces (n = 448), caeca (n = 220), colorectum (n = 99), duodenum (n = 99), ileum (n = 559), jejunum (n = 99), and crop (n = 40), totaling 1,514 samples across all gut regions.

**
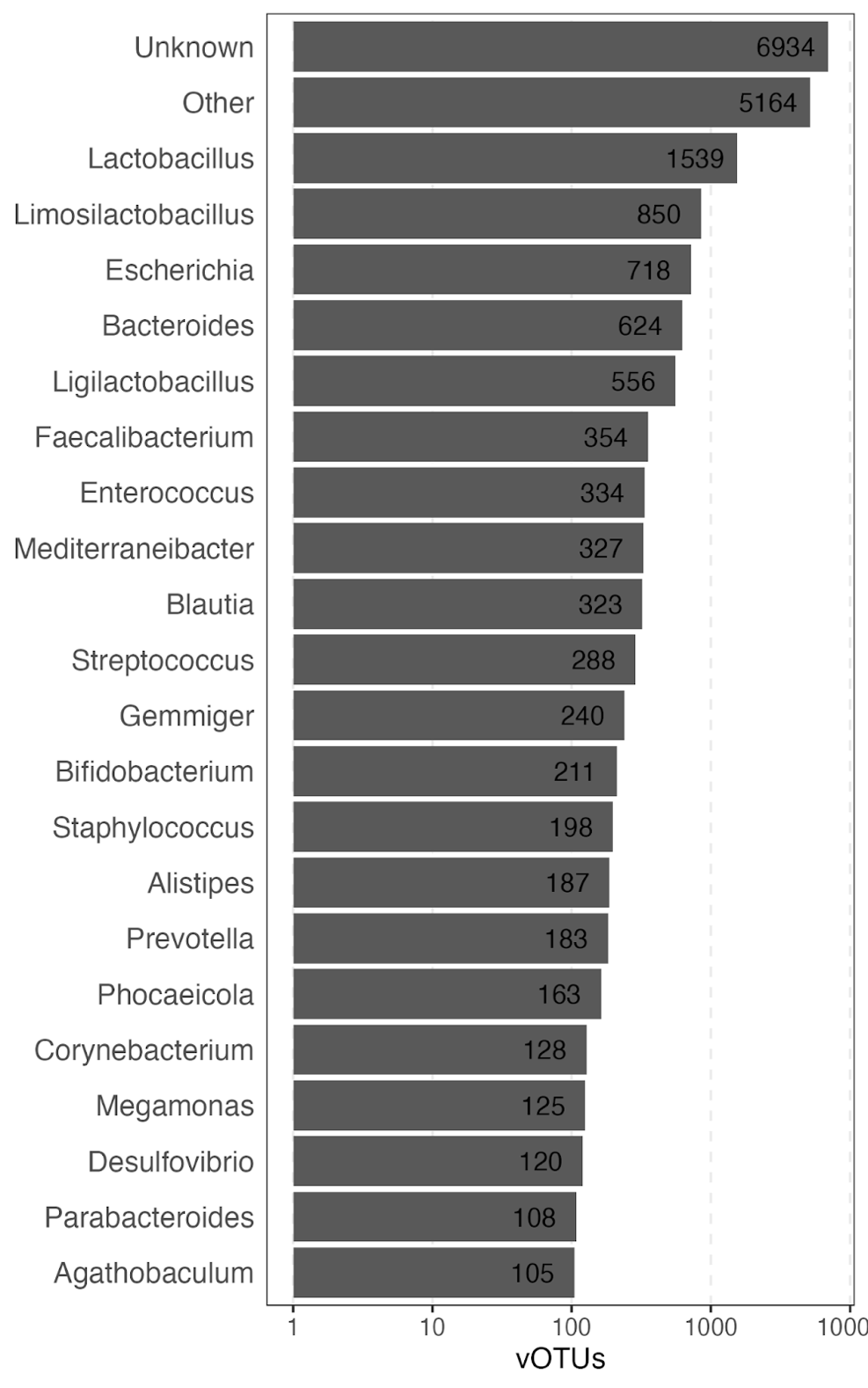
**

**Figure S5. Number of species-level vOTUs linked to a host, at genus levels, using Integrated Phage HOst Prediction (iPHoP).**

**
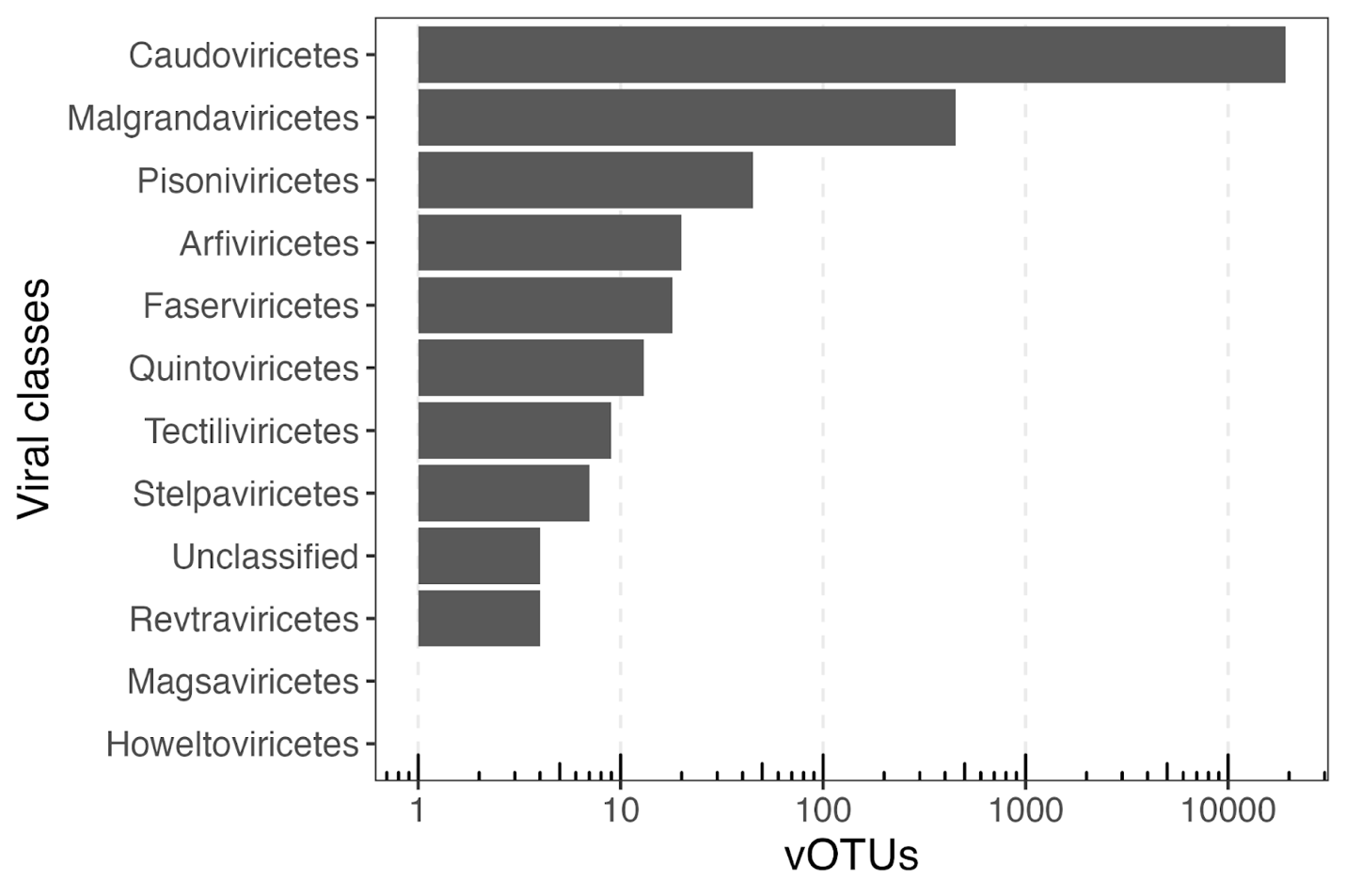
**

**Figure S6. Number of species-level vOTUs by predicted viral class. X-axis is depicted in logarithmic scale.**


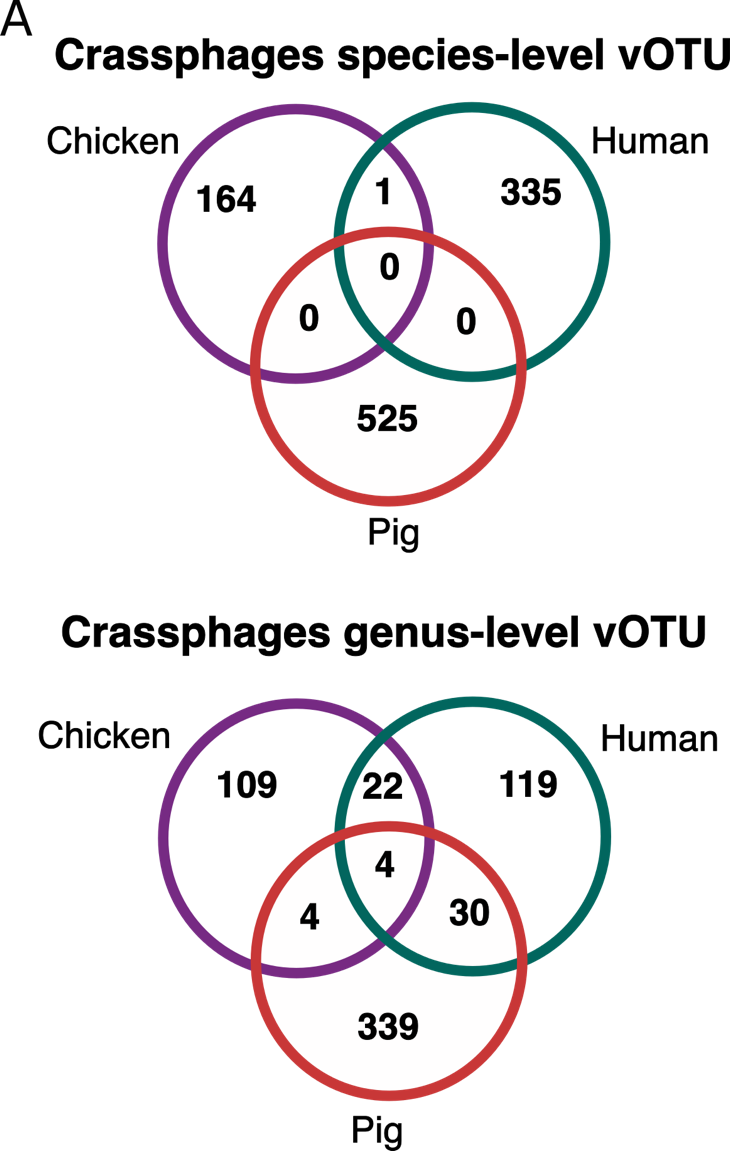


**Figure S7. Number of Crassvirales species-level vOTU and genus-level vOTU shared between chicken, human and pigs.**
